# An essential malaria protein defines the architecture of blood-stage and transmission-stage parasites

**DOI:** 10.1038/ncomms11449

**Published:** 2016-04-28

**Authors:** Sabrina Absalon, Jonathan A. Robbins, Jeffrey D. Dvorin

**Affiliations:** 1 Division of Infectious Diseases, Boston Children's Hospital, Boston, Massachusetts 02115, USA; 2 Department of Pediatrics, Harvard Medical School, Boston, Massachusetts 02115, USA; 3 Division of Infectious Diseases, Massachusetts General Hospital/Brigham and Women's Hospital, Boston, Massachusetts 02115, USA

## Abstract

Blood-stage replication of the human malaria parasite *Plasmodium falciparum* occurs via schizogony, wherein daughter parasites are formed by a specialized cytokinesis known as segmentation. Here we identify a parasite protein, which we name *P. falciparum* Merozoite Organizing Protein (PfMOP), as essential for cytokinesis of blood-stage parasites. We show that, following PfMOP knockdown, parasites undergo incomplete segmentation resulting in a residual agglomerate of partially divided cells. While organelles develop normally, the structural scaffold of daughter parasites, the inner membrane complex (IMC), fails to form in this agglomerate causing flawed segmentation. In PfMOP-deficient gametocytes, the IMC formation defect causes maturation arrest with aberrant morphology and death. Our results provide insight into the mechanisms of replication and maturation of malaria parasites.

## Abstract (web summary)

Blood-stage malaria parasites replicate through a specialised type of cell division known as schizogony. Here, Absalon *et al*. identify a parasite protein that is essential during schizogony for cytokinesis and formation of the inner membrane complex, the structural scaffold of daughter parasites.

## Graphical abstract

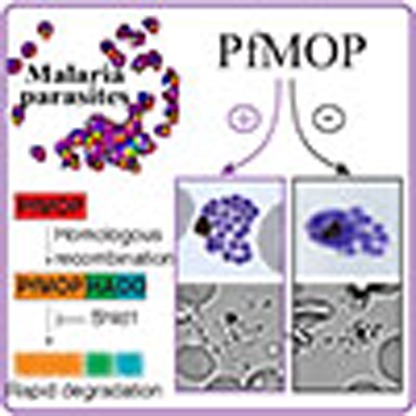

P*lasmodium spp.* infections cause ∼200 million cases of malaria and 500,000 deaths annually, with the most severe forms caused by *Plasmodium falciparum*^1^. The complete parasite life cycle requires both the mosquito and human hosts (Supplementary Fig. 1). Clinical malaria results from asexual proliferation of parasites in human red blood cells^2^. These blood-stage parasites replicate via schizogony, wherein repeated nuclear divisions produce a multi-nucleated cell. Individual nuclei and associated organelles are partitioned to produce daughter parasites during a specialized cytokinesis known segmentation, which is divergent from cellular division in the human host^3^. Because the process of cellular division is so different from human cells, an understanding of its molecular mechanism could reveal vulnerable targets for anti-malarials. The inner membrane complex (IMC), a specialized structure within the parasite composed of parasite proteins and a double lipid bilayer that is closely associated with the plasma membrane, is hypothesized to orchestrate parasite assembly and division^4^,^5^,^6^. In addition to its role in parasite division, the IMC plays a critical role in cellular architecture and gliding motility^7^,^8^,^9^,^10^,^11^. Two Rab-GTPases, Rab11a and Rab11b, are known to contribute to vesicular transport that is important for IMC formation^12^,^13^, but other factors that regulate IMC biogenesis remain largely unknown. During the blood-stage of human malaria, a subset of parasites differentiates into transmission forms, known as gametocytes, which are ingested during a mosquito blood meal. The IMC is central to the architecture of the developing gametocyte as well^10^,^14^. Parasite proteins important for regulation and biogenesis of the gametocyte IMC are also largely unknown in *P. falciparum*. A deeper understanding of the mechanism of blood-stage parasite division with particular focus on IMC formation will facilitate the discovery of novel anti-malarial therapeutics.

Here we show that PF3D7_0917000, which we have named *P. falciparum* merozoite organizing protein (PfMOP), is essential for the biogenesis of the IMC in both asexual and sexual parasites. Following PfMOP knockdown, blood-stage parasites undergo incomplete segmentation resulting in a residual agglomerate of partially divided cells. While parasite organelles develop normally, the IMC fails to form in this agglomerate. The IMC defect is more severe in the long-lived transmission stage where aberrant formation of the IMC in PfMOP-deficient gametocytes causes maturation arrest and death. These results show that PfMOP, through its regulation of IMC formation, is critical for the cellular architecture of both blood and transmission stages of human malaria.

## Results

### PfMOP is essential for replication of *P. falciparum* parasites

**Figure 1:**
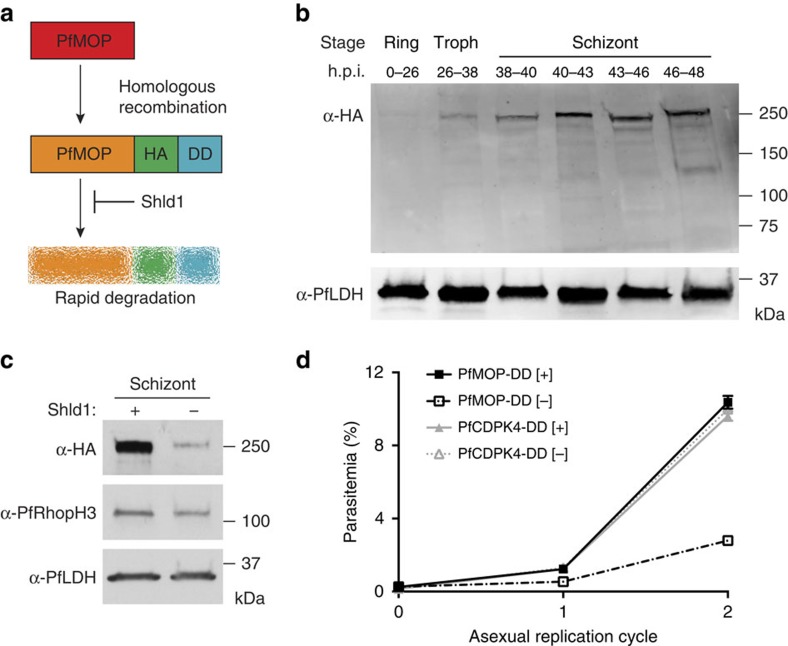
Generation of PfMOP-DD transgenic parasites. (**a**) Schematic of the DD system. PfMOP-DD-fusion protein is targeted for rapid degradation in the absence of Shld1. A haemagglutinin (HA) epitope tag is also present. (**b**) Protein lysates prepared from ring, trophozoite (Troph) or schizont stage PfMOP-DD parasites cultured with 250 nM Shld1 and probed with antibodies to HA or PfLDH (loading control). (**c**) Immunoblot of protein lysates from PfMOP-DD schizont stage parasites (36–48 h.p.i.) cultured [+]/[−] 250 nM Shld1 and probed with antibodies to HA, PfRhopH3 (a schizont stage loading control) or PfLDH (loading control). Quantification of immunoblot was performed by volumetric measurement of fluorescence intensity with the LiCor Odyssey CLx system. (**d**) Replication curves of PfMOP-DD and PfCDPK4-DD parasites cultured [+]/[−] 250 nM Shld1 (*n*=3, mean with ±s.d. error bars).

While investigating the mechanisms of parasite egress^15^, we discovered a conserved 1826 amino acid protein of unknown function, PF3D7_0917000 (hereafter named PfMOP), which has orthologs in other *Plasmodium spp*. (Supplementary Fig. 2)^16^,^17^. PfMOP contains an Armadillo repeat motif, but no signal peptide or transmembrane domains. Importantly, unlike the Armadillo repeat motif-containing PfARO (or TgARO in *Toxoplasma gondii*)^18^,^19^, PfMOP has no myristoylation signal. Multiple attempts to knockout PfMOP (by double crossover recombination^20^) were unsuccessful, strongly suggesting that PfMOP is essential for asexual replication of *P. falciparum*. By genetically fusing the destabilization domain (DD)^15^,^21^,^22^,^23^,^24^ to the carboxy-terminus of PfMOP, we generated 3D7-PfMOP-DD parasites, allowing inducible regulation of endogenous protein levels (Fig. 1a and Supplementary Fig. 3). DD-fusion proteins are stabilized in the presence of the ligand Shield-1 (Shld1) and degraded in its absence. 3D7-PfMOP-DD parasites can be cultivated without growth defect in the presence of Shld1. Agreeing with transcriptional profiling^16^, PfMOP is expressed primarily during the schizont stage (Fig. 1b). To evaluate PfMOP knockdown, ring-stage 3D7-PfMOP-DD parasites were maintained in the presence or absence of 250 nM Shld1 until the late schizont stage, and immunoblot demonstrated 60% knockdown following Shld1 removal (Fig. 1c).

**Figure 2:**
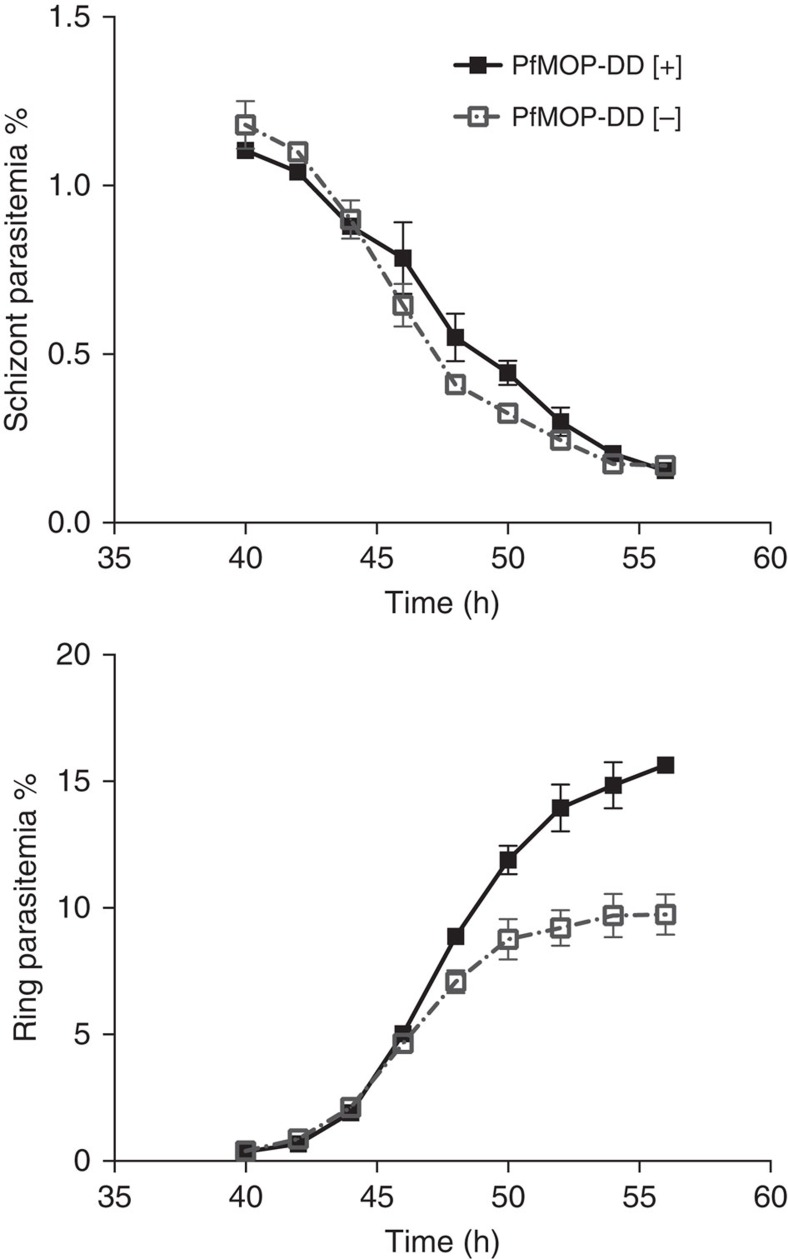
PfMOP is required for efficient asexual parasite growth. Time course of PfMOP-DD parasites cultured [+]/[−] Shld1. Samples were collected every 2 h from 40 to 60 h.p.i. and parasitemia was determined by flow cytometry (*n*=3, mean with 95% CI error bars).

To determine the requirement for PfMOP during asexual parasite development, we monitored replication of 3D7-PfMOP-DD parasites in the presence or absence of Shld1. As a control, we used the transgenic 3D7-PfCDPK4-DD parasites containing an inducible knockdown in PfCDPK4, a kinase which is not required for asexual parasite replication^15^,^25^,^26^. Over two asexual cycles, PfMOP-deficient parasites demonstrated a 73% decrease in parasitemia, while the control parasites show no decrease in growth (Fig. 1d). Within a single asexual cycle from ring-stage to newly re-invaded ring-stage, the replication defect is dose-dependent on the Shld1 concentration (Supplementary Fig. 4a,b). By light microscopy, 3D7-PfMOP-DD parasites with or without Shld1 develop from early ring-stages to multi-nucleated schizonts without loss of parasitemia within the development cycle. After washing away Shld1 from ring-stage parasites, the replication defect can be fully rescued by re-addition of the stabilizing ligand up until 38 h post invasion (h.p.i.), suggesting that PfMOP function is not required before this point (Supplementary Fig. 4c). To determine whether PfMOP-deficient parasites have alterations in the timing or extent of egress and invasion, we measured schizont rupture and ring formation by flow cytometry. The kinetics of schizont rupture were similar in the presence or absence of Shld1 (Fig. 2), thus molecular function of PfMOP is independent of the parasite egress signal.

### PfMOP-deficient parasites have defective segmentation

**Figure 3:**
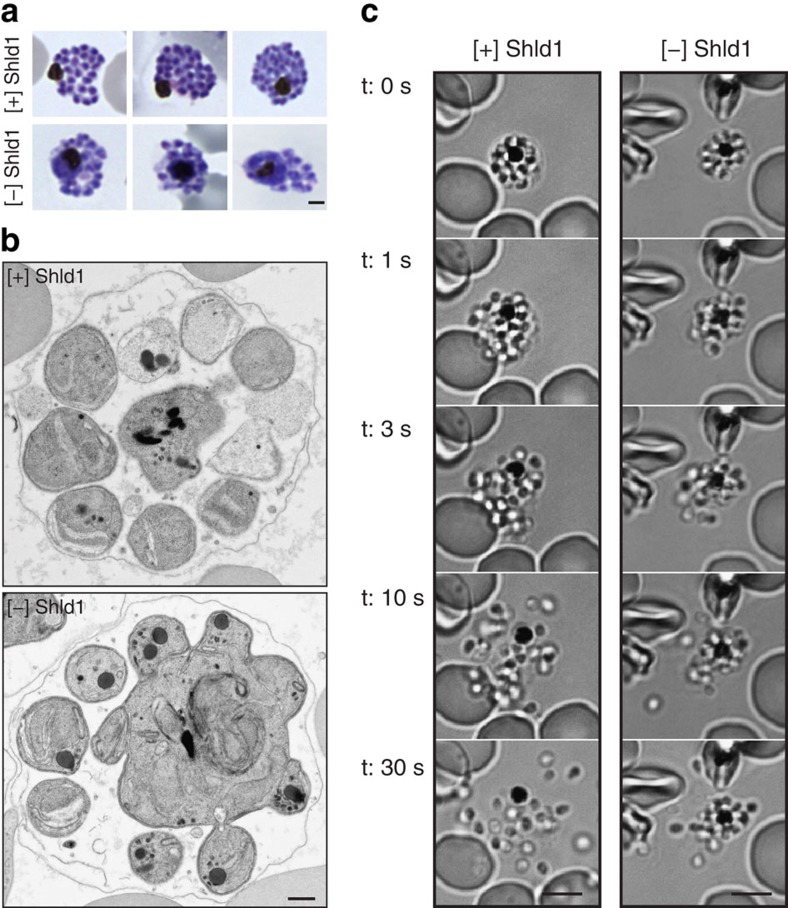
PfMOP is essential for schizont segmentation. PfMOP-DD parasites were grown [+]/[−] Shld1 until 52 h.p.i. (E64 added for 6 h) and visualized either by light microscopy ((**a**) scale bar, 1 μm) or by electron microscopy ((**b**) scale bar, 500 nm). (**c**). Frames from video microscopy of [+]/[−] Shld1 PfMOP-DD schizont rupture. The first frame with a released merozoite was arbitrarily set at *t*=1 s. Scale bar, 5 μm. Representative video shown for each condition. Nine rupture events scored for [+] Shld1 with 8 out of 9 having normal egress. Sixteen rupture events scored for [−] Shld1 with 1 out of 16 having normal and 15 out of 16 having aberrant egress.

During schizogony, parasites undergo multiple sequential rounds of asynchronous nuclear divisions^27^. At 44 h.p.i., schizonts maintained with and without Shld1 from the early ring-stage were treated with 10 μM E64, a cysteine protease inhibitor, to prevent release of daughter parasites while allowing the development of viable merozoites^28^,^29^. By treating late-stage schizonts with E64, ‘post-egress-trigger' parasites can be evaluated^30^. After 4 h of additional incubation, parasites were evaluated by light microscopy. In the absence of Shld1, E64-treated 3D7-PfMOP-DD parasites displayed an aberrant agglomerate of unsegmented merozoites surrounding the food vacuole (Fig. 3a). We quantified this effect by counting the number of fully segmented merozoites at different Shld1 concentrations. 3D7-PfMOP-DD parasites had, on average, 22.5 (22.2-22.9; 95% CI), 15.8 (13.4-18.2; 95% CI) and 11.0 (9.1-12.9; 95% CI) fully segmented merozoites per schizont when maintained with 250, 10 and 0 nM Shld1, respectively (Supplementary Fig. 5). Notably, the schizont parasitemia was similar for all Shld1 conditions. Thus, the major cell biological defect in PfMOP-deficient parasites is in segmentation. To reveal the ultra-structure of this incomplete budding, we compared E64-treated schizonts with and without Shld1 by electron microscopy (Fig. 3b). In the presence of Shld1, we observed distinct merozoites enclosed by a single membrane. In absence of Shld1, only few merozoites were separated from each other and a multi-nucleated agglomerate of merozoites was present. These experiments establish that PfMOP-deficient parasites have a developmental defect during the final stages of schizont segmentation.

Segmentation is a dynamic process. To better characterize the role of PfMOP in segmentation, we performed live video microscopy of late-stage parasites maintained in the presence or absence of Shld1. In [+] Shld1 cultures, schizonts underwent typical swelling^31^,^32^ before an explosive rupture with release of free merozoites into the supernatant (Fig. 3c and Supplementary Movie 1). By 28 s after rupture, all merozoites from [+] Shld1 parasites were freely mobile and separated from the residual body. The PfMOP-deficient schizonts ruptured with similar timing. However, a cluster of merozoites remained attached to the residual body (Fig. 3c and Supplementary Movie 2). Additionally, multiple merozoites that were free from the residual vacuole remained partially attached to each other.

### PfMOP localizes to apical area of parasites

**Figure 4:**
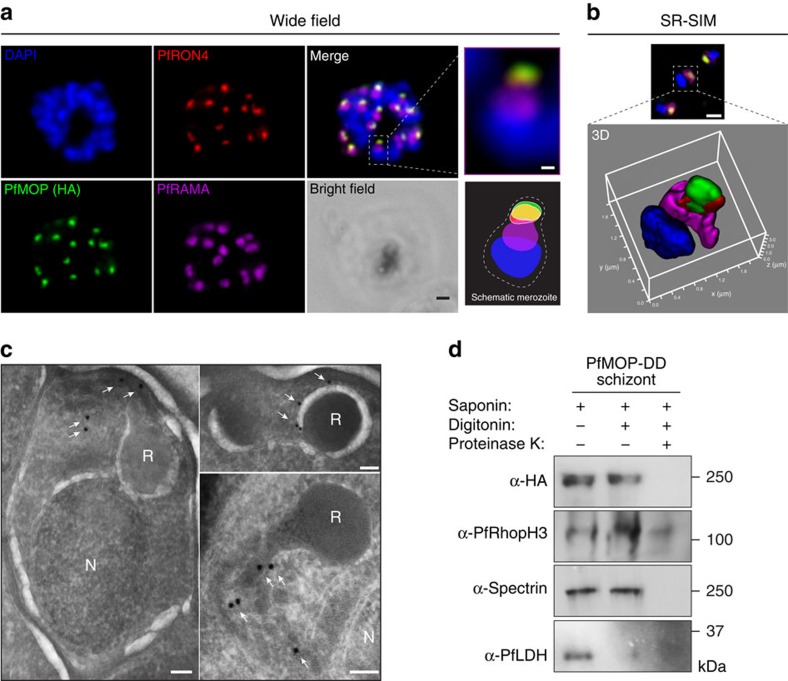
PfMOP localizes to apical area of parasites. Representative pictures of E64-treated schizont-stages. (**a**) Wide-field IFA of segmented schizont stained with antibodies against PfRAMA, PfRON4, and HA. Scale bar, 1 μm. (**b**) SR-SIM showing deconvolution of single z-section and three-dimensional reconstruction. Scale bar, 1 μm. (**c**) Transmission electron microscopy with (white arrows) gold-labelled antibodies against HA (N, nucleus; R, rhoptry, scale bar, 100 nm). (**d**) Proteinase K protection assay. Schizont stage parasites were permeabilized with saponin and [+]/[−] digitonin, [+]/[−] treated with proteinase K and probed with antibodies to HA. Control proteins are luminal rhoptry protein (PfRhopH3), cytoskeleton protein (spectrin) and cytosolic protein (PfLDH).

To study the molecular function of PfMOP, we evaluated its localization by immunofluorescence assays (IFA; Supplementary Fig. 6). Co-staining with markers directed against the endoplasmic reticulum (PfBiP), Golgi (PfERD2), apicoplast (PfBCCP) and micronemes (PfEBA175) did not reveal significant overlap with PfMOP (Supplementary Fig. 7). However, co-staining with rhoptry markers demonstrated similar localization to PfMOP. While approximate colocalization is present with the rhoptry bulb marker, PfRAMA, and the rhoptry duct marker, PfRhopH3 (ref. 33), the closest overlap was present with the rhoptry neck marker, PfRON4 (Fig. 4a). To visualize the localization of PfMOP in three dimensions, we performed super-resolution structured illumination microscopy (SR-SIM). This analysis demonstrated that PfMOP closely approximates the rhoptry neck, although its localization was not entirely overlapping with PfRON4 (Fig. 4b). PfMOP does not have a recognizable signal sequence or rhoptry targeting sequence^34^, thus this localization was unexpected. Immuno-electron microscopy revealed staining near the electron-dense rhoptries, consistent with the IFA (Fig. 4c). To biochemically characterize the localization of PfMOP, we performed a proteinase protection assay^18^. Following digitonin treatment to release soluble cytoplasmic proteins from the parasite, PfMOP remained present in the fractionated pellet. However, after treatment with Proteinase K, which digests proteins exposed on the cytoplasmic side of organelles, PfMOP is degraded (Fig. 4d), while the control rhoptry luminal protein, PfRhopH3, is partially protected. We further evaluated the localization of PfMOP by treating parasites with Brefeldin A (BFA), a small molecule that inhibits transport of vesicles out of the endoplasmic reticulum. While trafficking of the merozoite surface protein-1 (PfMSP1) is altered by BFA treatment, PfMOP localization remained largely unchanged (Supplementary Fig. 8). Thus, PfMOP is not trafficked through the classic secretory system.

### PfAMA1 trafficking is aberrant in the agglomerate

**Figure 5:**
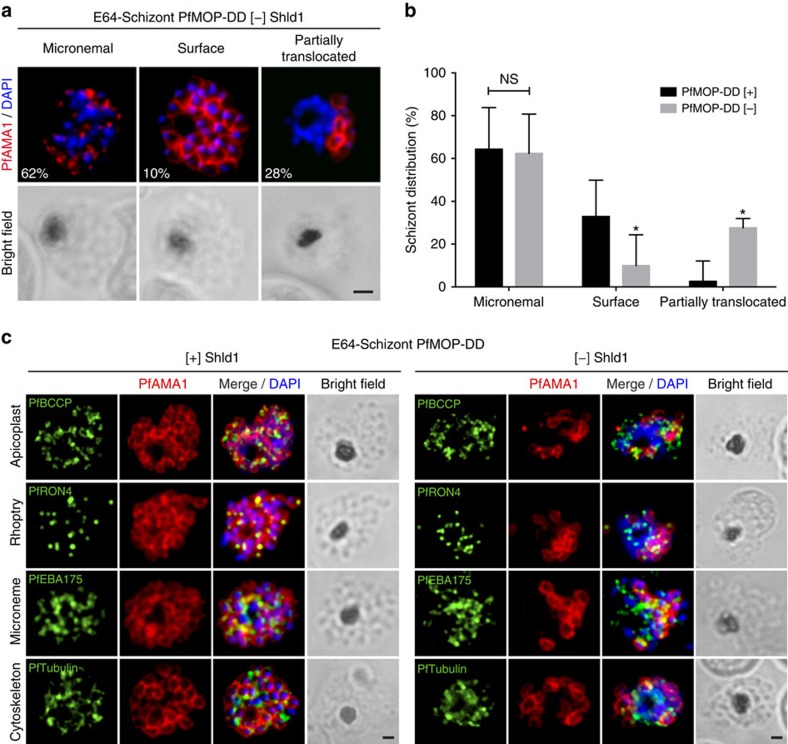
PfAMA1 translocation is aberrant in schizonts with PfMOP-knockdown. (**a**) Schizonts from [+]/[−] Shld1 PfMOP-DD parasites were E64-treated, fixed, probed with anti-PfAMA1 and scored as micronemal (M), partially translocated (PT) or surface (S), representative [−] Shld1 parasite IFAs shown. (**b**) Chart shows proportions of each type (100 schizonts per condition from *n*=3 independent experiments; mean with 95% CI error bars; *P<0.01, groups compared by unpaired *t*-test, scale bar, 1 μm). (**c**) Synchronized schizont stage (40–44 h) parasites, maintained with 250 nM (left panel) or 0 nM (right panel) Shld1, were incubated 6 h in presence of 10 μM E64, methanol-fixed, permeabilized, and stained using antibodies against PfRON4, PfRhopH3, PfEBA175 and PfTubulin. Staining for these markers was similar in [+] and [−] Shld1 conditions. PfAMA1 staining was used to identify E64-treated schizonts that were sufficiently mature (that is, surface staining or partially translocated staining, but not micronemal staining, scale bar, 1 μm).

In PfMOP-deficient parasites, some merozoites are fully segmented and invade normally (Fig. 2). During normal egress, the invasion ligand PfAMA1 switches localization from the apical microneme organelles to the plasma membrane of the newly released merozoites^30^,^35^. To evaluate the release of PfAMA1 from the PfAMA1-containing micronemes, we monitored localization by IFA. Furthermore, we used this relocalization to define the time of the ‘egress trigger' in schizonts by IFA in E64-treated 46±2 h.p.i. schizonts. By IFA, we only observe the agglomerate in parasites where the ‘egress trigger' has occurred. In [+] Shld1 parasites, 64% of schizonts had PfAMA1 localized as punctate micronemal staining (in parasites that had not been triggered), and 33% displayed plasma membrane localization (in schizonts where the ‘egress trigger' has occurred; Supplementary Fig. 9). While PfAMA1 was micronemal in 62% of PfMOP-deficient parasites, we observed uniform plasma membrane staining in only 10% of parasites, and a third aberrant category of staining in 28% (Fig. 5a,b). In the aberrant category, where the agglomerate is clearly present, parasites demonstrated surface staining on fully segmented merozoites and loss of signal in the multi-nucleated agglomerate. We note that under the conditions of this assay, 64% of the plus Shld1 and 62% of the minus Shld1 parasites, those with apical PfAMA1 staining, have not yet triggered and, in the PfMOP-knockdown parasites, the agglomerate has not yet formed.

We additionally evaluated the presence of PfRON4 (rhoptry marker), PfEBA175 (general microneme marker), PfBCCP (apicoplasts marker) and PfTubulin (cytoskeletal marker) in schizonts with PfMOP-knockdown. In parasites with apical PfAMA1 staining, those that had not passed the ‘egress trigger' time point, localization of these additional intracellular structures was similar in the plus and minus Shld1 conditions. Focusing on parasites where PfAMA1 had translocated, the localization of PfRON4, PfEBA175, PfBCCP and PfTubulin was also unchanged between plus and minus Shld1 parasites (Fig. 5c). These data demonstrate that while the PfAMA1-containing micronemes release their contents when triggered, PfAMA1 is not trafficked properly in the agglomerate. However, this defect is not likely due to a general absence of other intracellular structures in the agglomerate.

### PfMOP is involved in IMC formation

**Figure 6:**
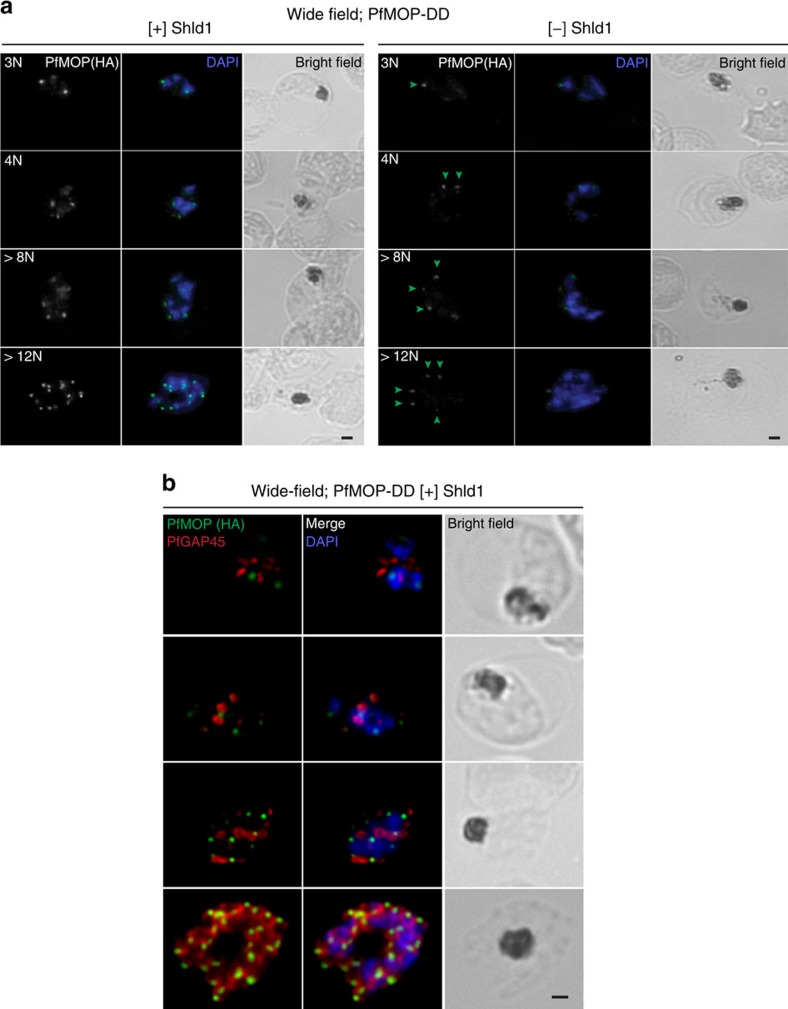
Residual PfMOP in knockdown parasites forms fewer and less bright accumulations. (**a**) Synchronized parasites maintained [+] and [−] Shld1 were sampled for processing every 2–4 h from 36 to 44 h.p.i., methanol-fixed, stained with the anti-HA antibody and counterstained with DAPI. Green arrowheads indicate the dimmer foci of PfMOP staining in [−] Shld1 samples. The number of nuclei is shown in upper left corner. (**b**) Synchronized parasite cultured with Shld1 were fixed and probed with DAPI, anti-HA antibody (green) and anti-GAP45 antibody (red). Images are representative of multiple independent biological replicates. Scale bar, 1 μm.

The IMC has been hypothesized to serve as a scaffold for daughter parasite formation in Apicomplexan organisms^4^,^5^,^6^. As PfMOP-DD in [−] Shld1 conditions results in a knockdown (but not knockout), we were able to follow residual MOP throughout schizogony in parasites with and without PfMOP-deficiency (Fig. 6a). In [+] Shld1 parasites, even before apical organelles have formed, PfMOP localizes to an area of newly forming daughter merozoites. In [−] Shld1 parasites, there are fewer, less intense punctate PfMOP spots in the apical end of a subset of developing merozoites. Notably, PfMOP staining is absent from the agglomerate. Thus in PfMOP-knockdown parasites, segmented merozoites have detectable PfMOP by IFA, whereas in the residual unsegmented agglomerate PfMOP is not detected. These data suggest that PfMOP may play a role in defining the apical end of the forming merozoite, perhaps directing the formation of the nascent IMC.

To follow the formation of the IMC, we evaluated the localization of PfGAP45, a protein component of the IMC, in early time points during schizogony (Fig. 6b). In the early schizont stage with three nuclei, we already see expression of both PfGAP45 and PfMOP by IFA. At this stage, the two proteins appear as two distinct spots. As the development of the schizont progresses to the mid-schizont stage, the localization of PfGAP45 changes to form a small ring-like structure (as previously described^8^), while PfMOP remains punctate near these forming ring-like structures. In the late-stage schizont, when the IMC is nearly fully formed, PfMOP and PfGAP45 overlap at the apical end of the daughter merozoites.

**Figure 7:**
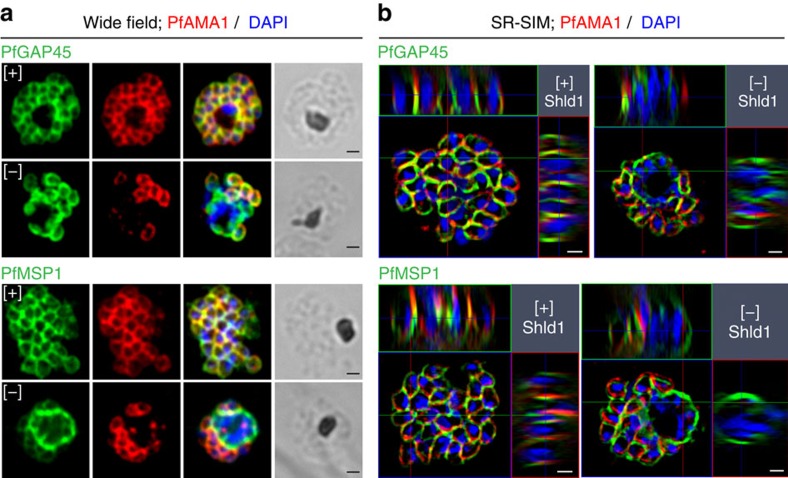
PfMOP deficiency leads to incomplete formation of the IMC. (**a**) Wide-field and (**b**) SR-SIM of representative pictures of E64-treated [+]/[−] Shld1 schizont stage PfMOP-DD parasites using antibodies against PfGAP45 and PfMSP1. Scale bar, 1 μm.

To test if PfMOP is critical for IMC formation, we evaluated the localization of PfGAP45 and PfMSP1, a marker for the parasite plasma membrane, in late-stage post-‘egress-trigger' parasites with and without PfMOP deficiency. Utilizing both wide-field microscopy (Fig. 7a) and SR-SIM (Fig. 7b), PfGAP45 and PfMSP1 displayed the expected pattern in [+] Shld1 parasites, with well-formed IMC and plasma membrane visible between each nucleus of the segmented schizont. However, in PfMOP-deficient parasites, PfGAP45 and PfMSP1 are missing from the agglomerate interior. Furthermore, serial z-sections with SR-SIM revealed no IMC or plasma membrane between multiple nuclei within the agglomerate (Fig. 7b). Thus, the major defect in PfMOP-deficient parasites is the failure of the IMC to form in the agglomerate, resulting in a failure of budding and the absence of plasma membrane enclosure of daughter merozoites. Evaluation of an additional IMC marker, PF3D7_0525800 (ref. 8), showed similar findings (Supplementary Fig. 10). These data support the hypothesis that PfMOP is critical, either directly or indirectly, for the formation of the IMC in maturing schizonts. Furthermore, our data provide direct experimental evidence that proper IMC formation is an integral process of merozoite segmentation.

### Released merozoites from knockdown schizonts invade normally

**Figure 8:**
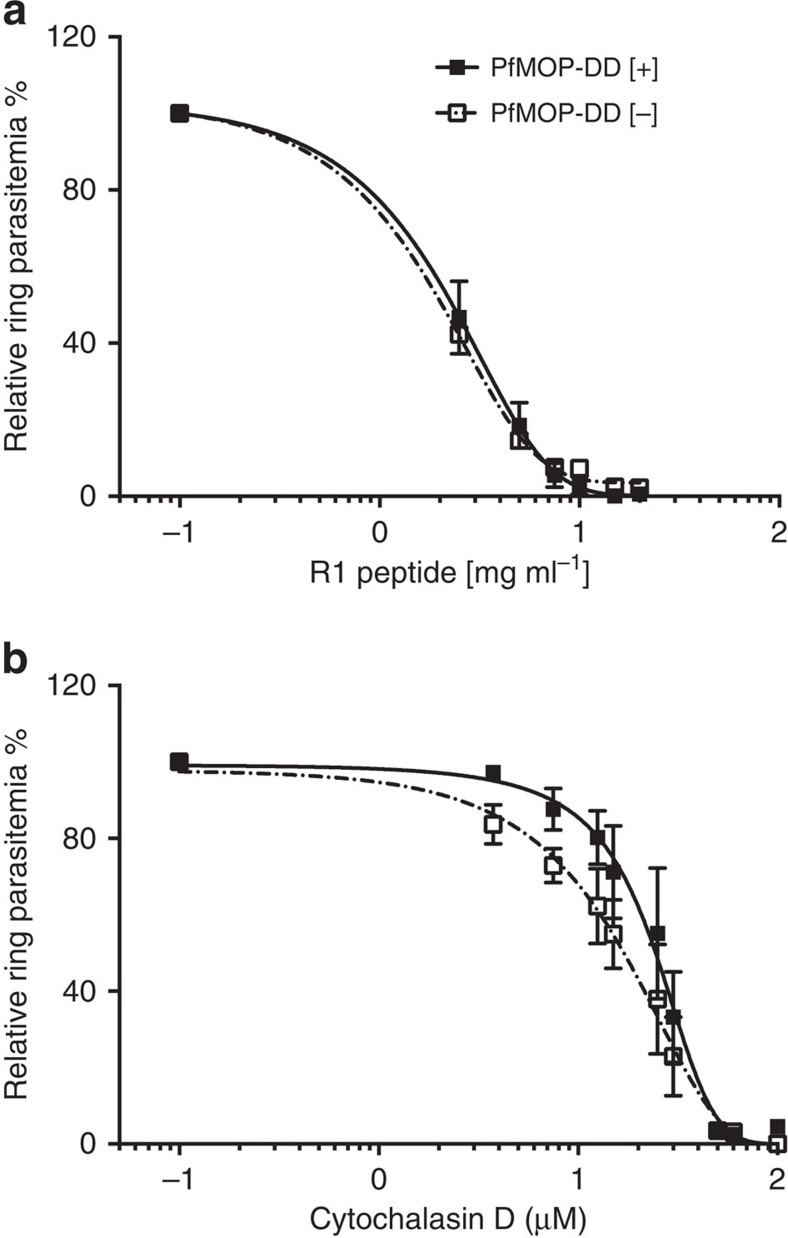
Released merozoites from PfMOP-deficient schizonts invade normally. Analysis of the sensitivity of PfMOP-DD parasites to inhibition of invasion by either the R1 peptide (**a**) or cytochalasin D (**b**). Synchronized schizont stage (42–46 h.p.i.) parasites, maintained [+]/[−] Shld1, were purified, then incubated for an additional 8–12 h with a range of R1 peptide or cytochalasin D concentrations. Newly re-invaded ring-stage parasites were measured by flow cytometry (R1 peptide *n*=3, cytochalasin D *n*=2, mean with 95% CI error bars, non-linear fit: log (agonist) versus response EC50).

*P. falciparum* invasion is a multistep process^36^,^37^. To evaluate invasion of merozoites released from schizonts with and without PfMOP knockdown, we compared the sensitivity to R1, a peptide that blocks tight junction formation between the parasite and the host red blood cell^38^,^39^, and to cytochalasin D, an inhibitor of actin polymerization that blocks the late actinomyosin-based invasion step^40^. Normalized to invasion with no inhibitor, sensitivity to R1 and cytochalasin D was similar in 3D7-PfMOP-DD parasites with and without Shld1 (Fig. 8, parental 3D7 shown in Supplementary Fig. 11). Thus, released merozoites from [−] Shld1 schizonts, where the bulk level of PfMOP has been reduced, invade normally. Because the PfMOP knockdown is not a knockout, we conclude that either PfMOP is not required for invasion or that the residual amount in released merozoites may be sufficient for any putative invasion-related function. To evaluate the release of invasion ligands from the apical organelles directly, we enzymatically treated infected cultures (in the presence or absence of Shld1) with trypsin, chymotrypsin and neuraminidase to prevent parasite reinvasion. The quantities of PfEBA175, a marker for microneme secretion, and PfRh2a, a marker for rhoptry secretion, were unaffected by the relative amount of PfMOP (Supplementary Fig. 12), indicating that apical organelle release was not generally inhibited in merozoites released from schizonts with PfMOP deficiency.

### PfMOP is essential for survival of gametocytes

**Figure 9:**
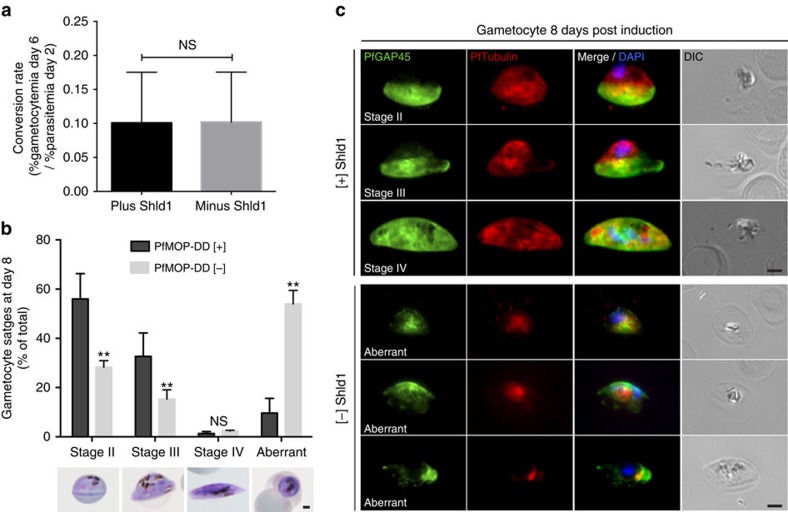
PfMOP is essential for gametocyte maturation. (**a**) Gametocyte conversion rate (*n*=3; mean with 95% CI error bars, groups compared with unpaired *t*-test). (**b**) Prevalence of *P. falciparum* gametocyte stages in PfMOP1-DD induced and grown [+]/[−] Shld1 by light microscopy (100 gametocytes were counted per condition from *n*=3 independent experiments; mean with 95% CI error bars; **P<0.001, groups compared with unpaired *t*-test, scale bar, 2 μm). (**c**) IFA using antibodies against PfGAP45 and PfTubulin show expected stages/morphology in [+] Shld1 and reveal absence of IMC development and abnormal shape in [−]Shld1 gametocytes, scale bar, 2 μm.

Transcription data demonstrate PfMOP expression in gametocytes, the transmission stage of the parasite^16^,^41^. Published RNA sequencing data show low level of expression in stage II gametocytes that increases in later the stages^41^. To test PfMOP function in gametocytes, we induced gametocyte formation in [+] and [−] Shld1 conditions and monitored development. Gametocyte conversion rate was similar [+] and [−] Shld1 (Fig. 9a). By day 8, the development and morphology of PfMOP-deficient gametocytes were abnormal (Fig. 9b). The IMC is critical for the architecture of the maturing gametocyte^10^. By IFA, we see a near absence of normal staining for PfGAP45 and PfTubulin in PfMOP-deficient gametocytes (Fig. 9c). Between day 8 and 12, when the [+] Shld1 gametocytes mature from stages II–V, PfMOP-deficient gametocytes fail to mature, with 85±8% displaying aberrant morphology and pyknosis/cellular death. These data provide clear evidence that PfMOP is critical for the transmission stages of *P. falciparum*. In PfMOP-deficient parasites, the IMC does not form properly and leads to a *bona fide* arrest of gametocytogenesis.

## Discussion

**Figure 10:**
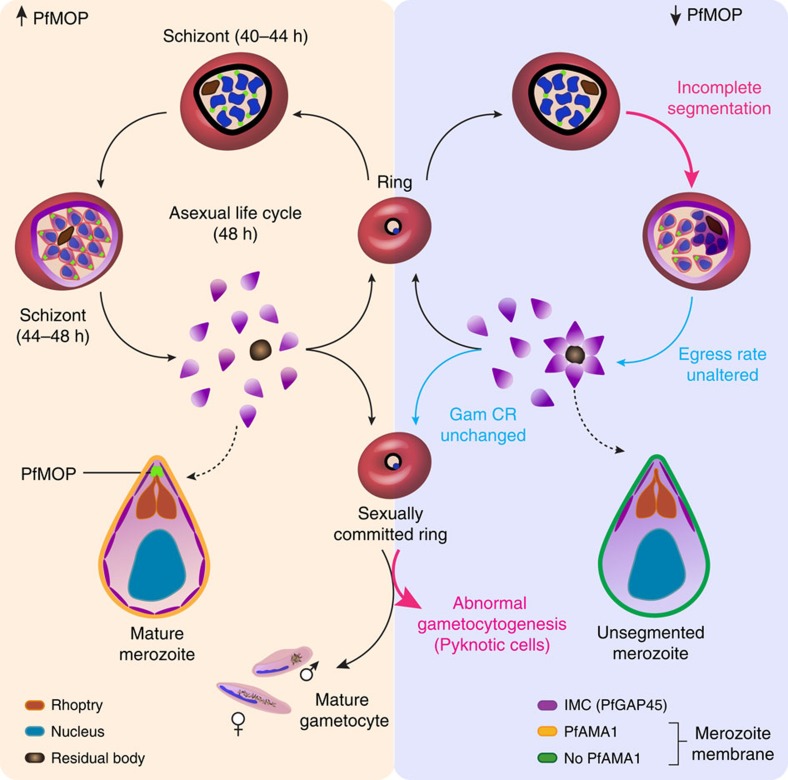
Model of PfMOP function in *Plasmodium falciparum* asexual life cycle. The schematic illustrates PfMOP protein localization and function through the asexual and transmission stages of the *P. falciparum* life cycle. In the left panel, the level of PfMOP protein is maintained by the presence of Shdl1. At the early schizont stage, parasites express PfMOP protein that localizes to the apical area of each newly forming daughter cell. As the schizont matures, PfMOP facilitates formation of the IMC, which leads to complete segmentation of the daughter merozoites through plasma membrane budding. For sexually committed rings, PfMOP expression facilitates IMC formation to maintain the survival and define the shape of the gametocyte. On the right panel, the consequences of PfMOP-deficiency are shown. At the early schizont stage, the quantity and number of PfMOP foci are both decreased. The low-level residual PfMOP is only detectable on a subset of forming daughter parasites, with the remaining trapped as an agglomerate under a common plasma membrane. In the absence of PfMOP, the IMC fails to form in the agglomerate. Egress is triggered normally, but the incompletely segmented merozoites remain attached to the residual body. The gametocyte conversion rate is unchanged by the absence of PfMOP. However, in absence of PfMOP, gametocytes lack an IMC and become aberrantly shaped and ultimately pyknotic.

Studies in *Toxoplasma gondii* and multiple *Plasmodium spp.* demonstrate that the IMC is critical to define the shape of the parasite, to anchor proteins for actinomyosin-based motility, and provide an architectural scaffold for newly formed daughter parasites^7^,^8^,^9^,^11^,^12^,^13^,^42^,^43^,^44^,^45^,^46^,^47^,^48^,^49^,^50^,^51^,^52^,^53^. Here we define the function of a previously uncharacterized protein that is critical for the biogenesis of the IMC in the *P. falciparum*, and we hypothesize a model of PfMOP function in Fig. 10. In late schizonts, PfMOP localizes to the apical end of daughter parasites. Interestingly, we demonstrate evidence of PfMOP expression in early schizonts (Fig. 6), likely before rhoptries or the IMC of the daughter parasites have begun to form. This finding suggests that PfMOP may be involved, directly or indirectly, in the early stages of IMC formation in daughter parasites.

The disrupted PfAMA1 localization observed in the agglomerate of triggered PfMOP-deficient schizonts (Fig. 5) suggests that IMC formation may be important for proper trafficking of PfAMA1 to the surface of the newly formed daughter merozoites. This finding potentially indicates a previously unrecognized role of the IMC, to ensure that PfAMA1 from the apical organelles reaches the surface of the released merozoite (or in E64-trapped ‘released' merozoites). As previously noted by Kono *et al*.^8^, the molecular mechanisms for trafficking of proteins to the IMC is not entirely understood. PfMOP does not have a recognizable signal sequence, transmembrane domain or rhoptry trafficking signal. Therefore, we hypothesize that the localization of PfMOP is controlled through interaction with other proteins, potentially on the cytoplasmic side of the rhoptry organelles. Two potential proteins include PfARO or PF3D7_0109000, the *P. falciparum* ortholog of TgPhIL1 (refs 18, 53, 54). The phenotype of PfMOP-deficient schizonts has some similarity to parasites treated with a specific inhibitor of phosphatidylinositol-4-OH kinase^55^. Interestingly, a subset of the parasites that were selected for resistance to these inhibitors had mutations in PfRab11a, implying that an underlying IMC defect may be present in inhibitor-treated parasites.

In PfMOP-deficient gametocytes, IMC formation failure is more severe, leading to death of the transmission stage (Fig. 9). Previously published RNA sequencing of asexual parasites and gametocytes demonstrate a lower level of PfMOP expression in early gametocytes compared with early schizonts^41^. This lower level of expression may allow for a more effective knockdown in early 3D7-PfMOP-DD gametocytes and thus explain the more severe phenotype. Fully segmented merozoites that are released from [−] Shld1 3D7-PfMOP-DD schizonts invade normally, thus demonstrating that they have a functional IMC. However, following invasion of sexually committed merozoites, the IMC is likely disassembled in the early stage gametocyte. When the IMC would normally form again after several days, there is insufficient PfMOP (having had the stabilizing agent washed out >5 days prior) to nucleate formation of the IMC. This result provides strong genetic evidence that the IMC is critical for survival of the transmission stages of *P. falciparum*. Anti-malarial therapeutics that disrupt IMC formation will likely be effective both in treating the disease-causing blood stages and in preventing maturation of transmission-stage gametocytes.

In conclusion, we have characterized PfMOP, a conserved protein that is essential in the blood stages of *P. falciparum* replication. PfMOP will provide a new starting point to understand the process of schizogony and IMC biogenesis.

## Methods

### Reagents and antibodies

Primers were obtained from Integrated DNA Technologies or Life Technologies; restriction enzymes were obtained from New England Biolabs. Commercially available antibodies were obtained from Roche Applied Science (rat anti-HA (3F10)), Life Technologies (mouse anti-HA (clone 2-2.2.14), Clontech (Rabbit anti-DsRed2) and Sigma-Aldrich (mouse anti-tubulin (clone B-5-1-2)). Other antibodies were kindly provided by Robin Anders at The Walter & Eliza Hall Institute of Medical Research (mouse anti-PfAMA1 clone 1FG; mouse anti-PfRESA, clone 28/2), Sean Prigge at Johns Hopkins Malaria Research Institute (rabbit anti-BCCP), Alan Cowman, Jenny Thompson and Kaye Wycherley at The Walter & Eliza Hall Institute of Medical Research (rabbit anti-PfEBA175, mouse anti-PfRON4), Dave Richards at Université Laval (mouse anti-PfRON4), Julian Rayner at Wellcome Trust Sanger Institute (rabbit anti-PfGAP45, rabbit anti-Rh2a), Anthony Holder at MRC National Institute for Medical Research (mouse anti-MSP1, clone 1E1), Ross Coppel at Monash University (Rabbit anti-PfRAMA), Odile Puijalon at Institut Pasteur Paris (mouse anti-PfRophH3) and Michael Makler at Flow Inc (mouse anti-PfLDH). Rabbit anti-PfBiP (MRA-19) and rabbit anti-ERD2 (MRA-1) were obtained through the Malaria Research and Reference Reagent Resource Center as part of the BEI resources, National Institute of Allergy and Infectious Diseases (NIAID), National Institutes of Health (NIH), contributed by John Adams.

### Parasite culture and transfection

The 3D7 strain of *P. falciparum*, obtained from the Walter and Eliza Hall Institute (Melbourne, Australia), was maintained *in vitro* in human O+ erythrocytes at 2% haematocrit in RPMI-1640 (Sigma) supplemented with 25 mM HEPES 4-(2-hydroxyethyl)-1-piperazineethanesulfonic acid (EMD Biosciences), sodium bicarbonate (Sigma), 50 mg l^−1^ hypoxanthine (Sigma), and 0.5% Albumax (Invitrogen)^56^. Sorbitol-synchronized ring-stage parasites at 2% parasitemia were transfected with ∼100 μg plasmid DNA by electroporation. Following transfection, parasites were maintained with 0.25 μM Shld1 and stable single crossover parasites were selected by cycling on and off WR99210 (Jacobus Pharmaceutical Company), as previously described^57^. Individual transgenic clones were obtained by limiting dilution. To generate the 3D7-PfMOP-DD-PFE1285w-mCherry parasite line, 3D7-PfMOP-DD parasites were transfected and selected on blasticidin (while maintaining parasites on Shld1 and WR99210).

### Plasmid construction

All targeting sequences for PfMOP were PCR amplified from parasite genomic DNA. To target the Pf3D7_0917000 gene locus for homologous recombination, the 3′ end was PCR amplified (oJDD538 5′- TAGGCGGCCGCTATTTTTCAACGAACCAAGATGCGTGG -3′/oJDD524 5′- GATCTCGAGCTTCGAAACAACATTTGTATAACTAGTTCTTC -3′) and cloned into our 3HA-DD plasmid^23^ cut with NotI/XhoI to generate pSAB01. For attempted knockout of PF3D7_0917000, we generated pCC1-PfMOP for double homologous recombination into the coding region of the PF3D7_0917000 locus. The upstream targeting fragment was PCR cloned (oJDD819 5′- TAGCTTAAGGCATGTGCTTTACAAATTGATGTA -3′/oJDD820 5′- GATCCGCGGCTCATTAAACCTACTAAGATACAA -3′) into AflII/SacI sites, and the downstream fragment PCR cloned (oJDD828 5′- TAGCCTAGGCTTCTAGATTTAAAGAAAATAAATGAAC -3′/5′- GATCCATGGACTACCATCACTTTTATTATTTTCATC -3′ oJDD822) into the AvrII/NcoI sites of pCC1 (ref. 58). To generate the 3D7-PfMOP-DD parasite line expressing PFE1285w-mCherry (PF3D7_0525800) the coding sequence was PCR amplified from 3D7 gDNA using oJDD1785 5′- TAGGGATCCATGTGTTCTACAAATAAGAATTTAGCT -3′ and oJDD1786 5′- GATCCTAGGAGCATATGAACAGTAAAGATTTCTTTGGAACAT -3′ and cloned into pB-CAM-mCherry^8^ (generously provided by Tim-Wolf Gilberger at Bernhard Nocht Institute) cut with BamHI/AvrII to generate pSAB67.

### Southern blot analysis

Harvested genomic DNAs were prepared with QIAamp Blood Mini Kit (Qiagen) and digested with enzymes NotI, XhoI, HincII and AgeI-HF. Digested genomic DNAs were resolved on 0.8% agarose gel, transferred to GeneScreen Plus (Perkin Elmer), and hybridized with radiolabelled probe specific for PfMOP (NotI/XhoI fragment from pSAB01).

### Western blot analysis and Proteinase K protection assay

*P. falciparum* proteins were extracted using 0.2% saponin (or 0.03% saponin for Proteinase K assay) and pellets were resuspended in Laemmli sample buffer. The protein samples for Proteinase K protection assay were prepared as described previously^18^. For immunoblot evaluation of PfEBA175 and PfRh2a release, infected cultures were treated with chymotrypsin (1 mg ml^−1^), trypsin (1 mg ml^−1^) and neuraminidase (66.7 mU ml^−1^) to prevent reinvasion, supernatants were harvested after schizont rupture, and clarified by centrifugation. All protein samples were separated on 4–20% mini-Protean TGX gels (Bio-Rad) and transferred to nitrocellulose membranes using the Trans-blot Turbo transfer system. Membranes were probes with primary antibodies in the following dilutions: anti-HA (1:1,000), anti-PfRophH3 (1:2,000), anti-PfRON4 (1:1,000), anti-Spectrin (1:2,000), anti-PfLDH (1:2,000), anti-PfEBA175 (1:500), anti-PfRh2a (1:500) and anti-carbonic anhydrase-1 (1:2,000). Primary antibody binding was detected using secondary antibody conjugated to horseradish peroxidase (at 1:10,000 dilution, GE Healthcare) or with antibodies directly labelled with near-infrared dyes (at 1:10,000 dilution, LiCor; 800CW donkey anti-goat, 680LT donkey anti-mouse). Quantification of immunoblot data was performed using volumetric measurement of fluorescence intensity on a LiCor Odyssey CLx imager. Immunoblot images were cropped for presentation. Full-sized images for each immunoblot are presented in Supplementary Fig. 13.

### Live video microscopy

Live microscopy was performed as described previously^59^. Briefly, microscopy dishes (Ibidi) were coated with a 0.5-mg ml^−1^ concanavalin A solution. Synchronized parasitized erythrocytes were applied to coated dishes and allowed to adhere for 10 min at 37 °C before being washed four times with PBS. Warmed complete phenol-red-free RPMI was added and dishes were sealed. Brightfield images were obtained every 1–5 s for 30 min per well, alternating between [+] Shld1 and [−] Shld1 wells, using a Nikon Eclipse Ti inverted microscope with a × 60 objective and Andor Zyla 4.2 scientific CMOS camera. Temperature was maintained at 37 °C during imaging. Images were processed using NIS Elements software. Egress events were scored as aberrant if two or more merozoites remained attached to the residual body following rupture.

### Immunofluorescence assays

Immunofluorescence microscopy was performed on slides with ice-cold methanol-fixed *P. falciparum* parasites. Slides were kept in humid chamber during the entire process. Parasites were permeabilized with 0.1% Triton × 100 for 3 min and blocked with 3% bovine serum albumin (BSA) for 1 h at room temperature. Primary antibodies were incubated over-night in cold room in the following dilutions: anti-PfAMA1 (1:200), anti-PfBCCP (1:100), anti-PfBiP (1:200); anti-DsRed2 (1:50), anti-PfEBA175 (1:500), anti-PfERD2 (1:200), anti-PfGAP45 (1:500), anti-HA (1:50), anti-PfMSP1 (1:500), anti-PfRAMA (1:200), anti-PfRON4 (1:200), anti-PfRohpH3 (1:1,000) and anti-PfTubulin (1:100). Subsequently, cells were washed three times with PBS and incubated for 45 min with the AlexaFluor 488, 555 or 647 secondary antibodies (1:2,000, Molecular Probes). After removal of unbound antibodies with three PBS washes, slides were mounted with Vectashield containing DAPI (Vector laboratories Inc.) with coverslips and kept at 4 °C until evaluation. All wide-field images were obtained with a Nikon E800 epifluorescence microscope using a × 100 (oil) objective and images were captured using SPOT Imaging software and then processed using Adobe Photoshop. SR-SIM Z-stacks were captured using an ELYRA PS.1 microscope (Carl Zeiss Microscopy). The ELYRA was used with a × 100x (oil) objective and excitation wavelengths of 405, 488, 561 and 638 nm. SIM images were collected at 100–200 nm *z* axis steps, with five rotations of the structured illumination grid were carried out per channel. Resulting stacks were processed using default reconstruction parameters in ZEN 2012 Black software. For electron microscopy, parasites were prepared as previously described 15. For immuno-electron microscopy, rat anti-HA was used at 1:50 with gold-labeled secondary antibody.

### Flow cytometry analysis of parasite replication

Parasites were synchronized at the schizont stage by density centrifugation on 60% Percoll PLUS, incubated at 37 °C for 2–3 h with fresh erythrocytes, and then newly-invaded ring-stage parasites were obtained by 5% (w/v) sorbitol treatment. Cultures were plated in triplicate and incubated at 37 °C at a haematocrit of 2% in presence or absence of Shld1. For each time point, 100 μl of culture was plated in triplicate into a U-Bottom 96-well plate. All samples were fixed using 1% paraformaldehyde in Alsever's solution for 20 min at room temperature. Cells were washed twice and resuspended with 0.5% BSA–PBS solution. Cells were then incubated with 100 μl of 1:1,000 SYBR green I (Life Technologies) for 20 min at room temperature. Cells were washed with 0.5% BSA–PBS solution and resuspended in PBS. Flow cytometry data was collected using a MACSQuant VYB (Miltenyi Biotec Inc.) with an acquisition of 100,000 events per sample. Initial gating was carried out with unstained, uninfected erythrocyte to account for erythrocyte auto-fluorescence.

### Cytochalasin D and R1 peptide assays

Parasites were tightly synchronized as described above, plated in 10cm dishes, and incubated at 37 °C at a haematocrit of 2% in presence or absence of Shld1. When matured to the 40–44 h.p.i., schizont stage parasites were plated in triplicate into flat-bottom 96-well plate and incubated for an additional 8–12 h with either cytochalasin D (Sigma) ranging from 0 to 60 μM in DMSO or R1 peptide (‘VFAEFLPLFSKFGSRMHILK', synthesized by Biomatik) from 0 to 20 mg ml^−1^ in DMSO. The parasitemia was determined by flow cytometry as above.

### Brefeldin A treatment

Synchronized schizont stage (32 h.p.i.) parasites were treated with either 5 μM BFA (Fisher) or DMSO for 5 h. The localization of PfMOP protein was assayed by immunofluorescence using anti-HA and compared with a Golgi marker PfERD2. As positive control, we assayed the localization of the plasma membrane protein PfMSP1 using a primary anti-PfMSP1 antibody.

### Gametocyte induction assay

Ring-stage parasites ± Shld1 at 2% parasitemia were plated with 50% conditioned medium to a final 2% haematocrit. After 2 days, the newly invade ring parasitemia was determined using flow cytometry and 10 mM heparin (Sigma H3149) was added to prevent subsequent reinvasion of asexual stage parasites, allowing monitoring of gametocyte formation. Gametocytemia (day 6) and gametocyte morphology (day 8) was assayed by light microscopy of thin blood smear stained with Field's staining solution. Gametocyte conversion rate was calculated as gametocytemia on day 6 divided by ring parasitemia on day 2. IFAs were performed on day 8.

## Additional information

**How to cite this article**: Absalon, S. *et al*. An essential malaria protein defines the architecture of blood-stage and transmission-stage parasites. *Nat. Commun.* 7:11449 doi: 10.1038/ncomms11449 (2016).

## Supplementary Material

Supplementary Information Supplementary Figures 1-13

Supplementary Movie 1 Live video microscopy of egress of 3D7-PfMOP-DD parasites cultured with 0.25 μM Shld1.

Supplementary Movie 2 Live video microscopy of egress of 3D7-PfMOP-DD parasites cultured without Shld1, displaying PfMOP-knockdown phenotype.
