# Supplementary material for: An essential malaria protein defines the architecture of blood-stage and transmission-stage parasites
Source: Nat Commun. 2016 Apr 28;7:11449. doi: 10.1038/ncomms11449 (PMC4853479; doi:10.1038/ncomms11449)
Supplement: Supplementary Information — Supplementary Figures 1-13 [file ncomms11449-s1.pdf]

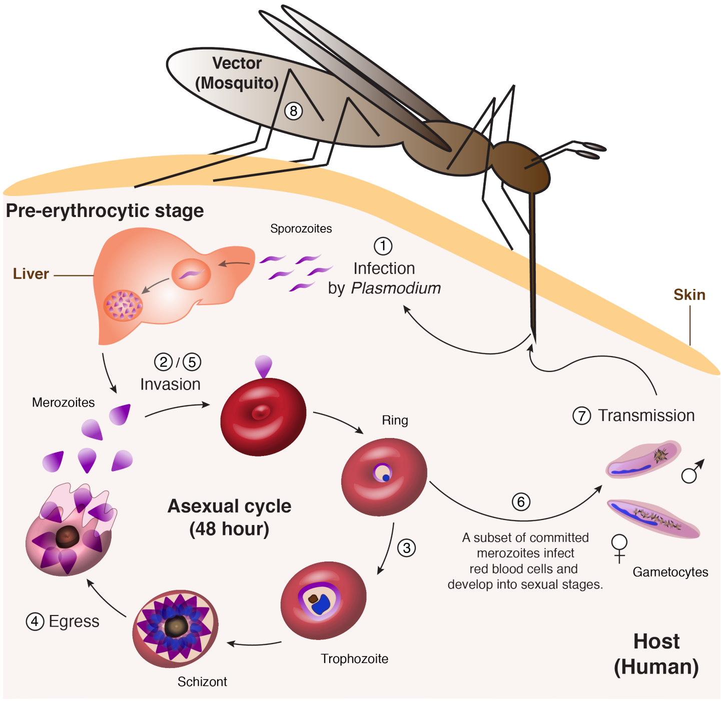

Supplementary Fig. 1. Schematic of *P. falciparum* life cycle.

Malaria parasites enter the human bloodstream in the form of sporozoites (1) after an infectious bite by a female *Anopheles spp* mosquito and then travel to the liver where they invade and multiply. Merozoites are released into the bloodstream and invade erythrocytes (2), initiating the exponential phase of the asexual life cycle. Parasites undergo multiple round of intra-erythrocytic development (3) progressing through ring, trophozoite, and schizont stages. Following egress (4), the newly released merozoites re-invade erythrocytes to repeat the intra-erythrocytic life cycle (5). A small fraction of schizonts release merozoites that are committed to differentiate into gametocytes following re-invasion (6). Gametocytogenesis takes 10 to 12 days to produce male and female gametocytes that are taken up by a mosquito during a blood meal (7). In the mosquito (8), the male and female gametocytes fuse to form a zygote. The zygote transform into an ookinete, then an oocyst, which release infectious sporozoites that travel to the mosquito salivary glands.

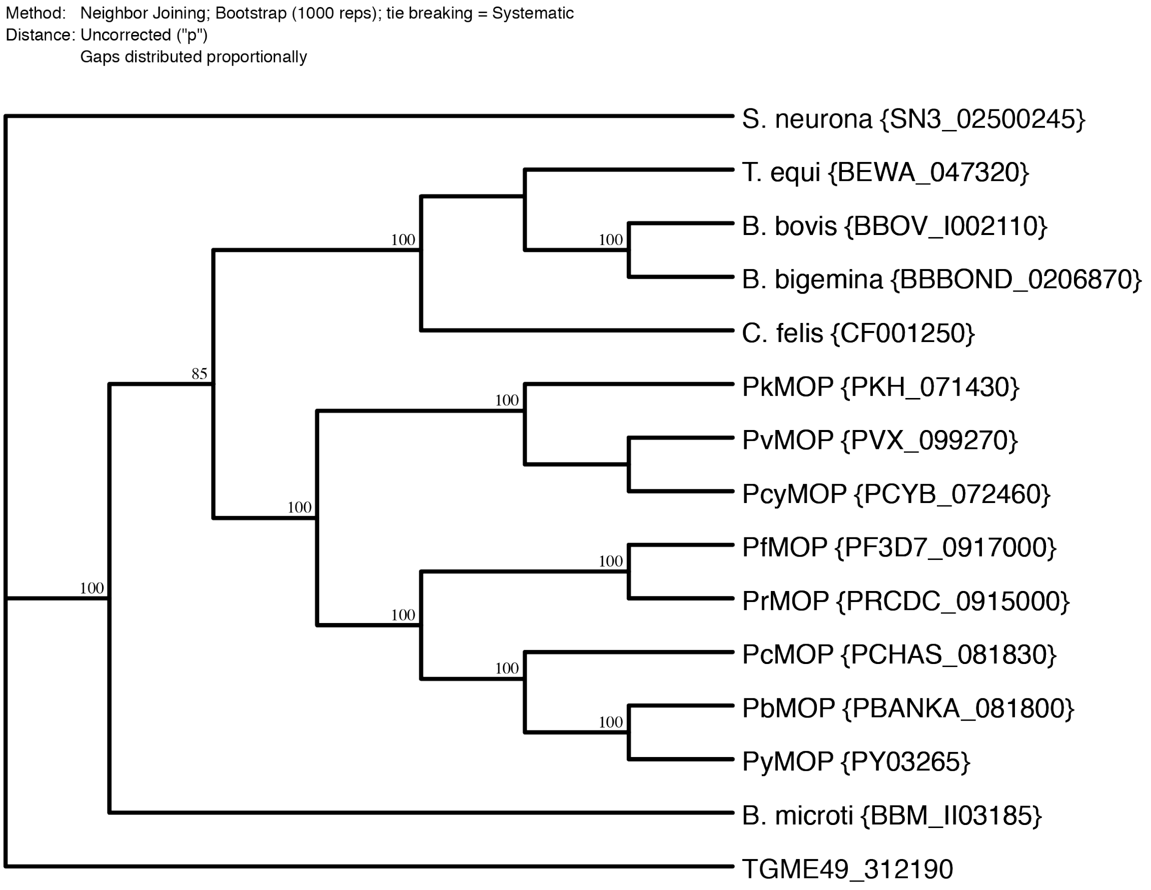

Supplementary Fig. 2. Bioinformatic analysis of PfMOP orthologs.

The primary protein sequence of PfMOP was used to identify orthologs in other eukaryotic organisms via BLASTP search against the entire Eupathdb.org database and a secondary search specifically against *Toxoplasma* and *Sarcocystis.* Potential orthologs were obtained from *P. reichenowi, P. knowlesi, P. vivax, P. cynomolgi, P. berghei, P. chabaudi, P. yoelii, B. microti, B. bovis, B. bigemina, T. equi, C. felis, S. neurona,* and *T. gondii*. The primary amino acid sequences were aligned using the T-coffee algorithm (via MacVector software). The phylogenetic tree was generated by neighbor-joining, bootstrap (1000 repetitions) methodology (via MacVector software). Phylogenetic tree reveals a closely related cluster within *Plasmodium spp.* with a second cluster within the piroplasms (*Babesia* and *Theileria*).

a

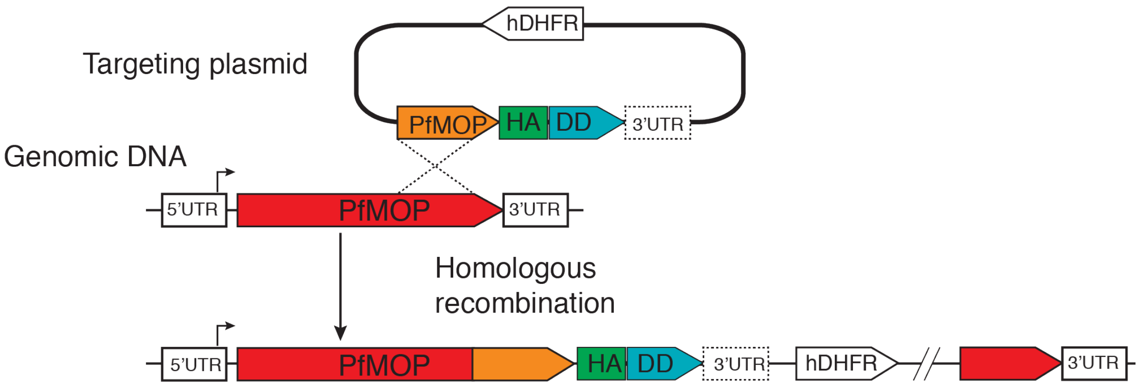

b

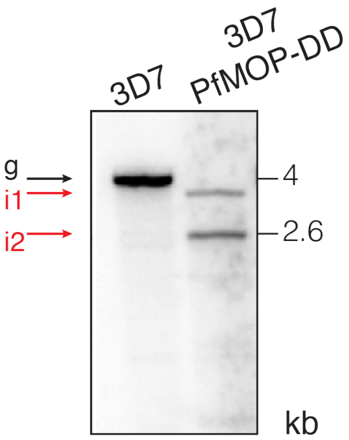

Supplementary Fig. 3. Generation of PfMOP-DD transgenic parasites.

(**a**). Schematic of single crossover targeting vector, not drawn to scale. Parasites were transfected with the targeting plasmid, maintained with 0.25 μM Shld1, and cycled on and off WR99210 selection. Following integration of targeting plasmid, transgenic parasites were clones by limiting dilution. (**b**). Genomic DNAs from 3D7 and the sub-clone of 3D7-PfMOP-DD were digested with AgeI/HincII/NotI/XhoI and probed with a PfMOP specific probe. Expected sizes for wild type and integrants are 3986 bp ('g') and 3555 bp ('i1') / 2670 bp ('i2').

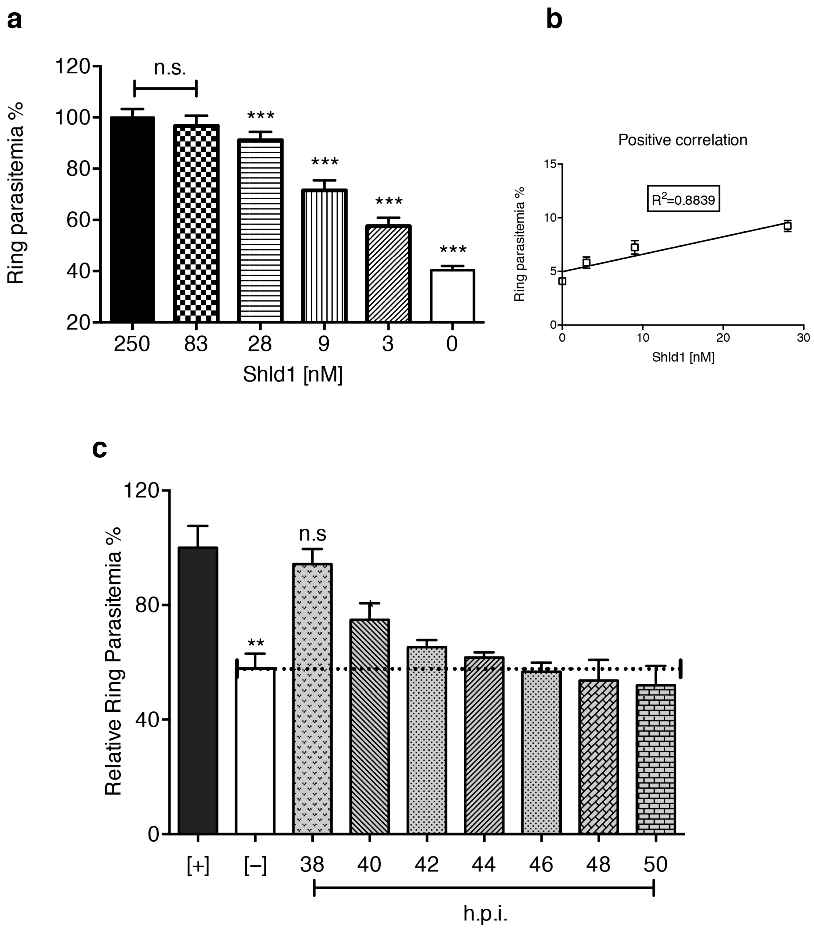

Supplementary Fig. 4. Correlation between Shld1 concentration and parasitemia of newly-invaded rings and Shld1 rescue.

(**a**). Representative parasitemia relative to Shld1 concentration. Synchronized ring-stage PfMOP-DD parasites were washed to remove Shld1, maintained for a complete asexual life cycle (>48 hours) with six different concentrations of Shld1, and resulting re-invaded relative parasitemia determined by flow cytometry (n=3, mean with 95% CI error bars, ***p<0.0001, groups compared by unpaired *t*-test). Ring-stage parasitemia at 250 nM Shld1 set to 100%. (**b**). Linear fit of % parasitemia relative to Shld1 concentration appears linear at lower concentrations of Shld1 (linear regression, 95% CI error bars). (**c**). Representative time course of the relative ring

parasitemia. Synchronized ring-stage PfMOP-DD parasites were washed to remove Shld1, maintained for a complete asexual life cycle (>48 hours) with re-introduction of Shld1 in the culture medium every two hours during schizogony (from 38 to 50 h.p.i), and resulting re-invaded relative parasitemia determined by flow cytometry (n=3, mean with 95% CI error bars, *p<0.001, **p<0.0001, groups compared by unpaired *t*-test).

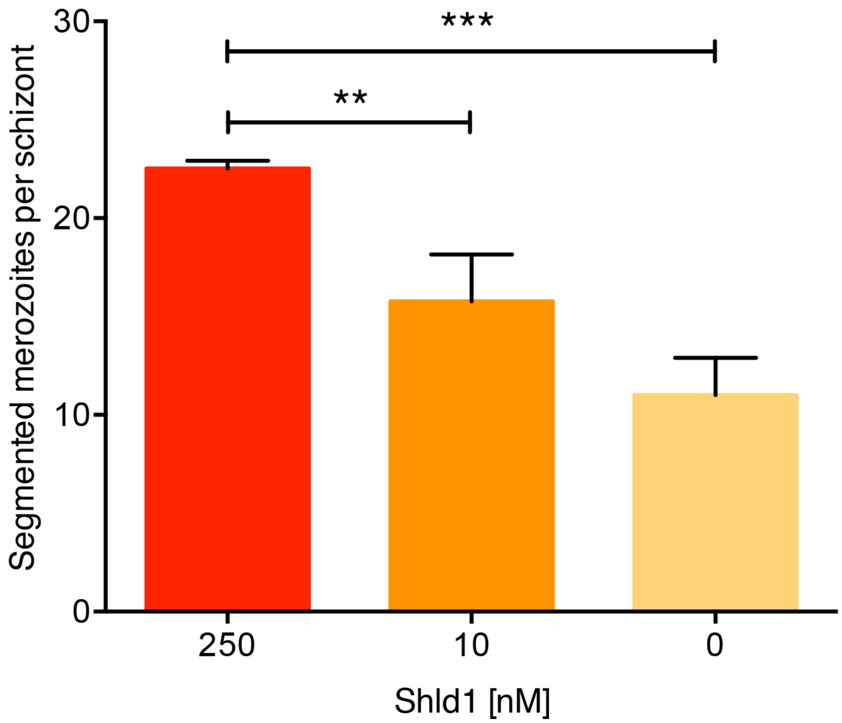

	250	10	0
Lower 95% CI of mean	22.15	13.40	9.106
Upper 95% CI of mean	22.91	18.15	12.91

Supplementary Fig. 5. The number of segmented merozoites per schizont is PfMOP1 dependent. Synchronized schizont-stage (40-44 h.p.i.) parasites maintained with 250, 10, or 0 nM of Shld1 were incubated six hours in presence of 10 μM E64, allowing counting of the fully segmented merozoites (separated from agglomerate) per schizont on a Field-stained thin blood smear (Approximately 15 schizonts were counted for each of n=3 independent experiments; mean with 95%CI error bars; ***$p<0.0001$, groups compared by unpaired *t*-test).

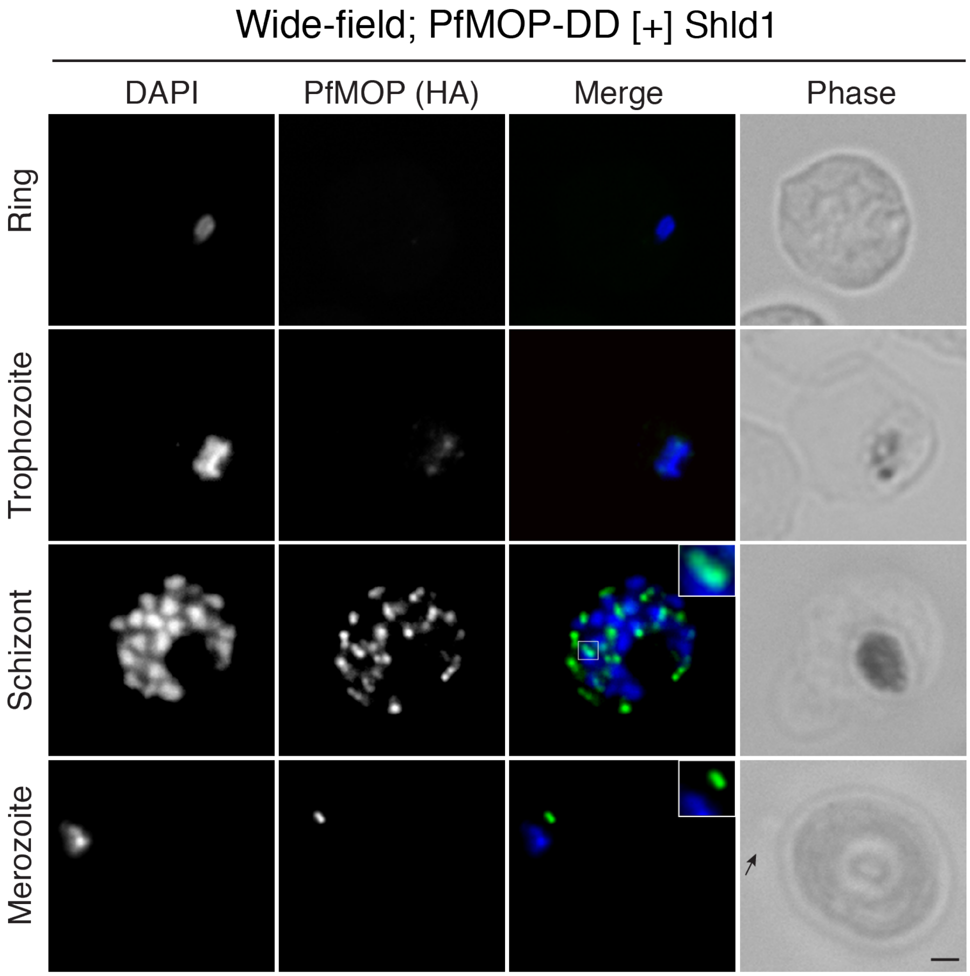

Supplementary Fig. 6. IFA of PfMOP throughout the asexual life cycle. IFA of asexual blood-stage PfMOP-DD parasites with antibodies against HA epitope within PfMOP-DD and counterstained with 4',6-diamidino-2-phenylindole (DAPI). PfMOP is seen in the apical regions of the schizont and within free merozoites. Enlargement of selected areas are marked with a white square. The black arrow indicates the position of the free merozoite. Scale bar is 1 µm.

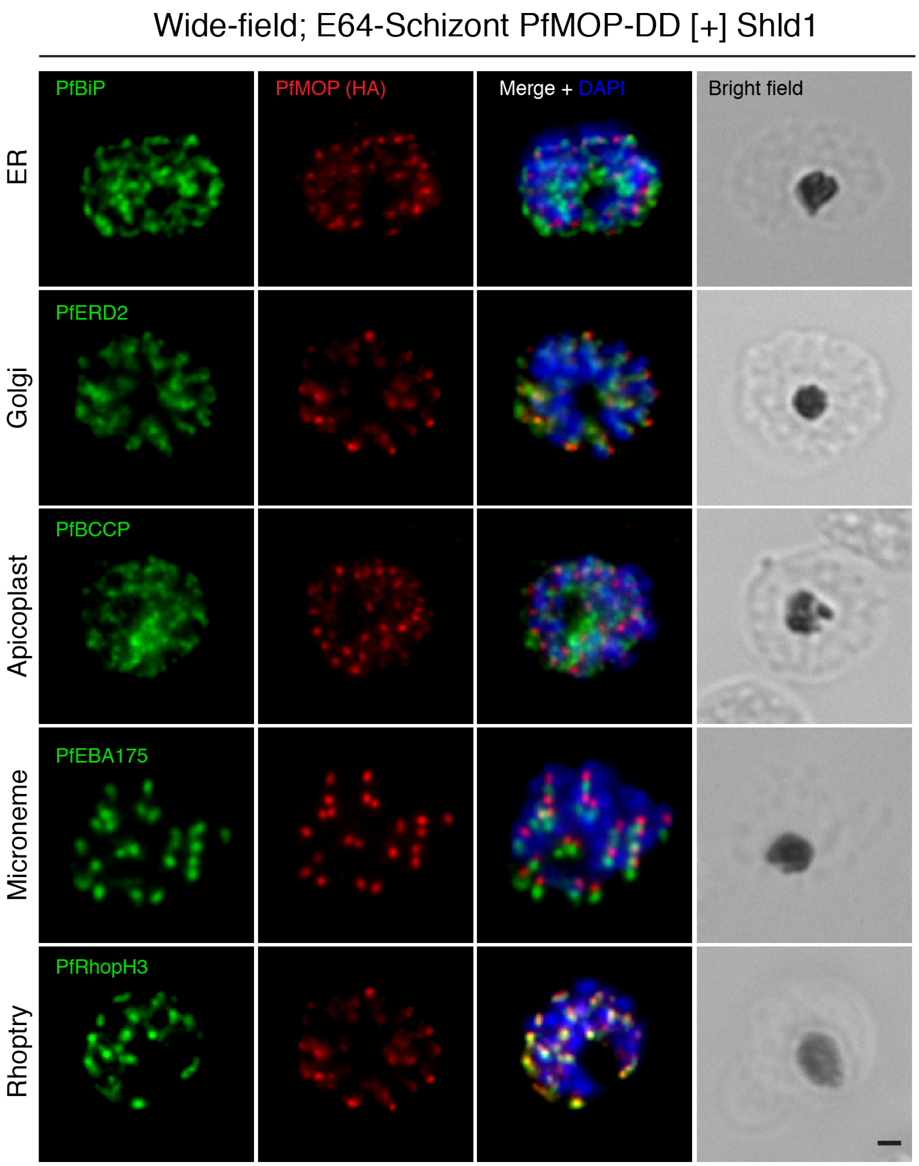

Supplementary Fig. 7. Co-localization IFAs of PfMOP in schizont-stage parasites.

Methanol-fixed, permeabilized, E64-treated schizonts maintained on 250 nM Shld1 were examined by IFA with the antibodies to proteins in the endoplasmic reticulum (PfBiP), Golgi (PfERD2), apicoplast (PfBCCP), rhoptries (PfRhopH3), or micronemes (PfEBA175), and counterstained with DAPI, as indicated. Images were obtained using a 100X lens on a NikonE800. Images are representative from several independent biological replicates, scale bar, 1 μm.

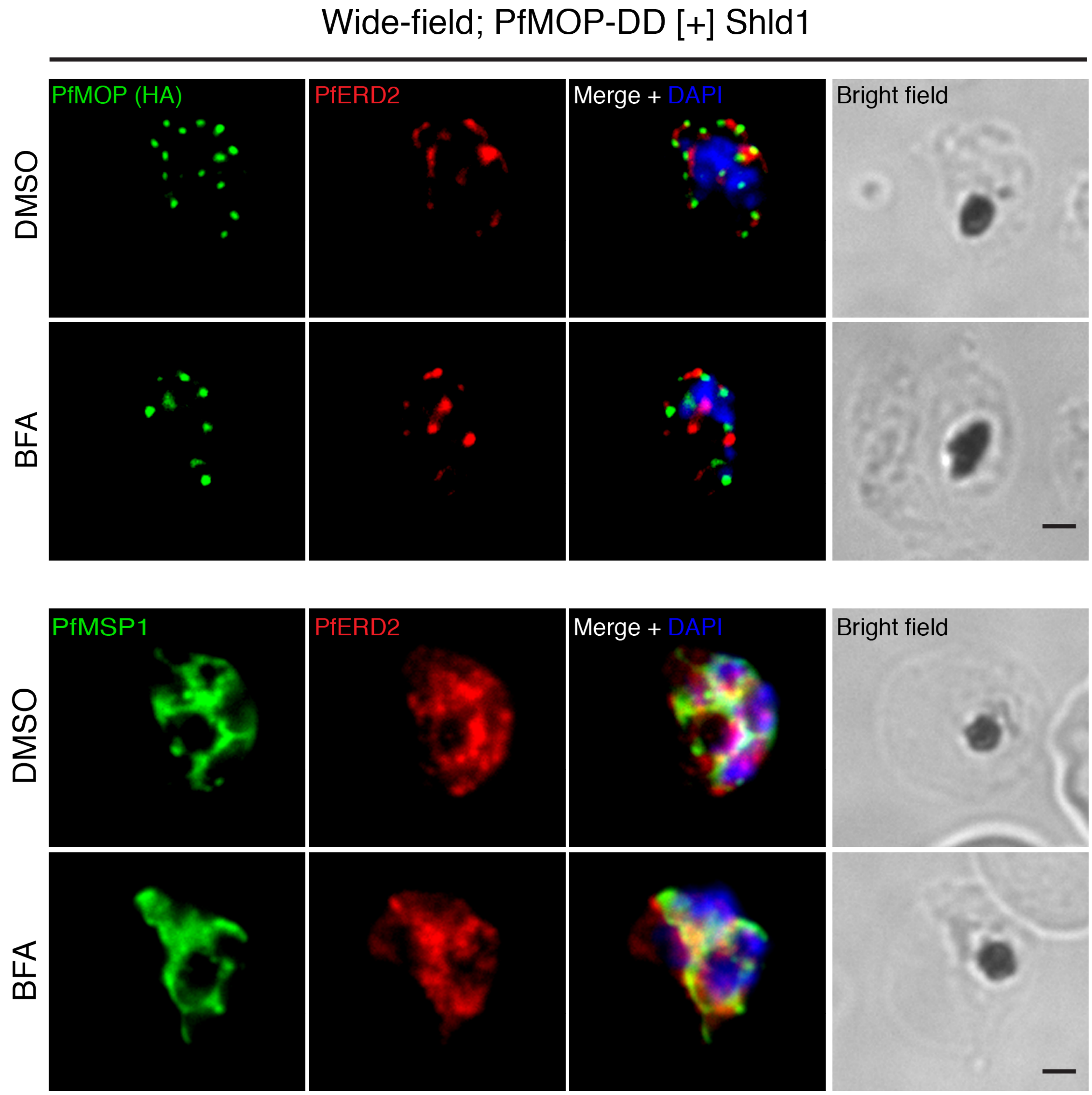

Supplementary Fig. 8. PfMOP localization following Brefeldin A (BFA) treatment. Synchronized, early schizont-stage (36-38 h.p.i.) parasites, maintained with 250 nM Shld1 were incubated for five hours with either 5 μM BFA or equivalent volume of a DMSO-only control. (**a**). PfMOP trafficking was assayed by IFA using antibodies against HA and a Golgi marker (PfERD2), scale bar, 1 μm. (**b**). As control of the BFA action, the same samples were processed with antibodies against a merozoite plasma membrane surface protein (PfMSP1) that is known to traffic through the secretory pathway, scale bar, 1 μm.

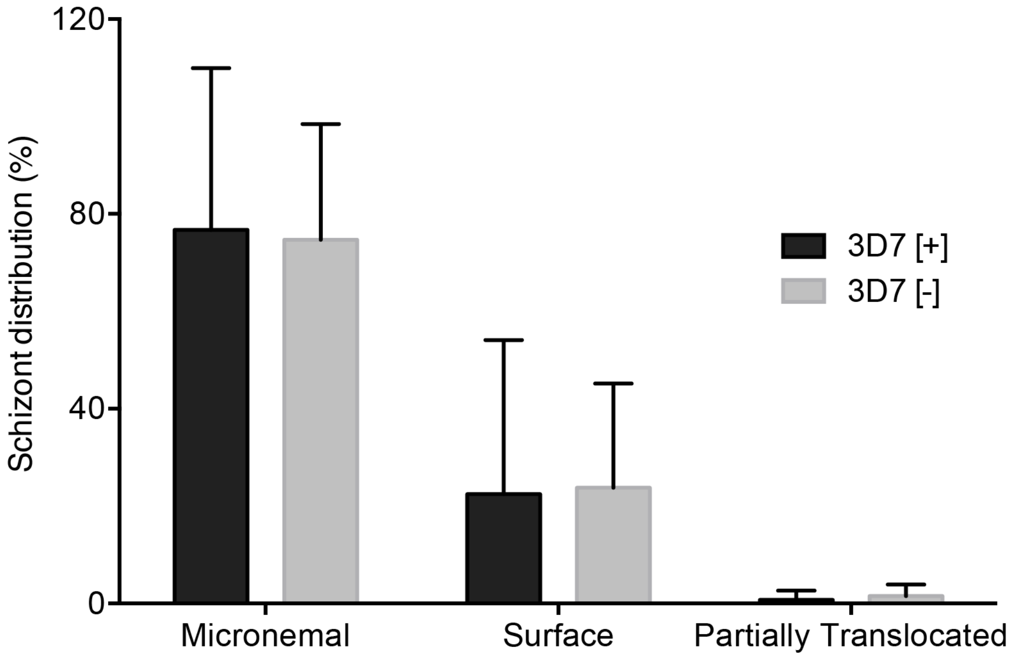

Supplementary Fig. 9. PfAMA1 translocation in 3D7 parental strain. Schizonts from [+]/[-] Shld1 3D7 control parasites were E64-treated, fixed, probed with anti-PfAMA1, and scored as micronemal (M), surface (S), or partially translocated (PT). Chart shows proportions of each pattern type. A minimum of 50 schizonts per slide per condition was counted (100 schizonts counted per condition from n=3 independent experiments; mean with 95%CI error bars; *$p<0.01$, unpaired *t*-test; differences between [+] and [-] Shld1 conditions were not significant, groups compared by unpaired *t*-test).

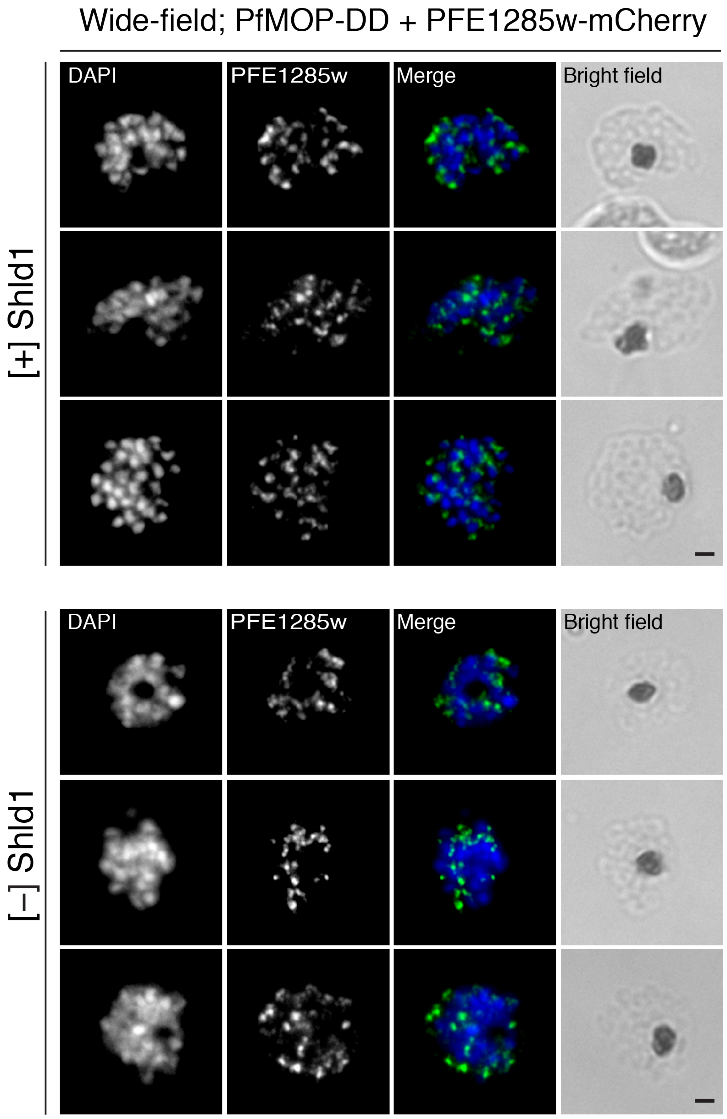

Supplementary Fig. 10. PFE1285W is absent from the agglomerate in PfMOP-deficient parasites. An episomal vector expressing PFE1285w (Pf3D7_0525800) fused to the mCherry fluorescent protein was transfected into 3D7-PfMOP-DD parasites. Synchronized parasites were maintained [+] and [-] Shld1 until 44-46 h.p.i., methanol-fixed, stained with antibodies against mCherry, and counterstained with DAPI. In [+]Shld1 parasites (top), PFE1285w staining is observed associated with each nucleus. In [-]Shld1 parasites, similar to the staining pattern observed for PfGAP45, the PFE1285w signal is lacking in the agglomerate. Scale bar is 1 μm.

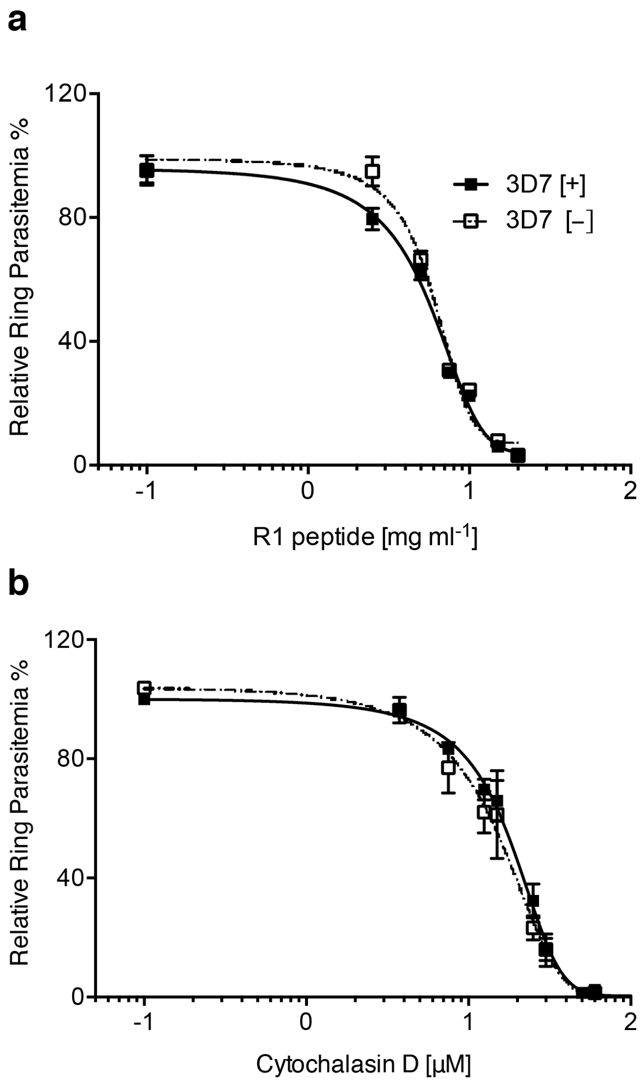

Supplementary Fig. 11. Wild type merozoite sensitivity to R1 peptide and cytochalasin D. (**a**). The sensitivity of the parental 3D7 parasites to inhibition of invasion by the R1 peptide was assayed. Synchronized schizont-stage (42-46 h.p.i.) maintained [+]/[-] Shdl1 were purified, then incubated for an additional 8-12 hours with a range of R1 peptide concentration. Newly re-invaded ring-stage parasites were measured by flow cytometry (n=2, mean with 95% CI errors bars, non-linear fit: Log(agonist) vs. response EC50)). (**b**). Cytochalasin D dose-response assays were performed as described in (**a**) above (n=2, mean with 95% CI errors bars, non-linear fit: Log(agonist) vs. response EC50)).

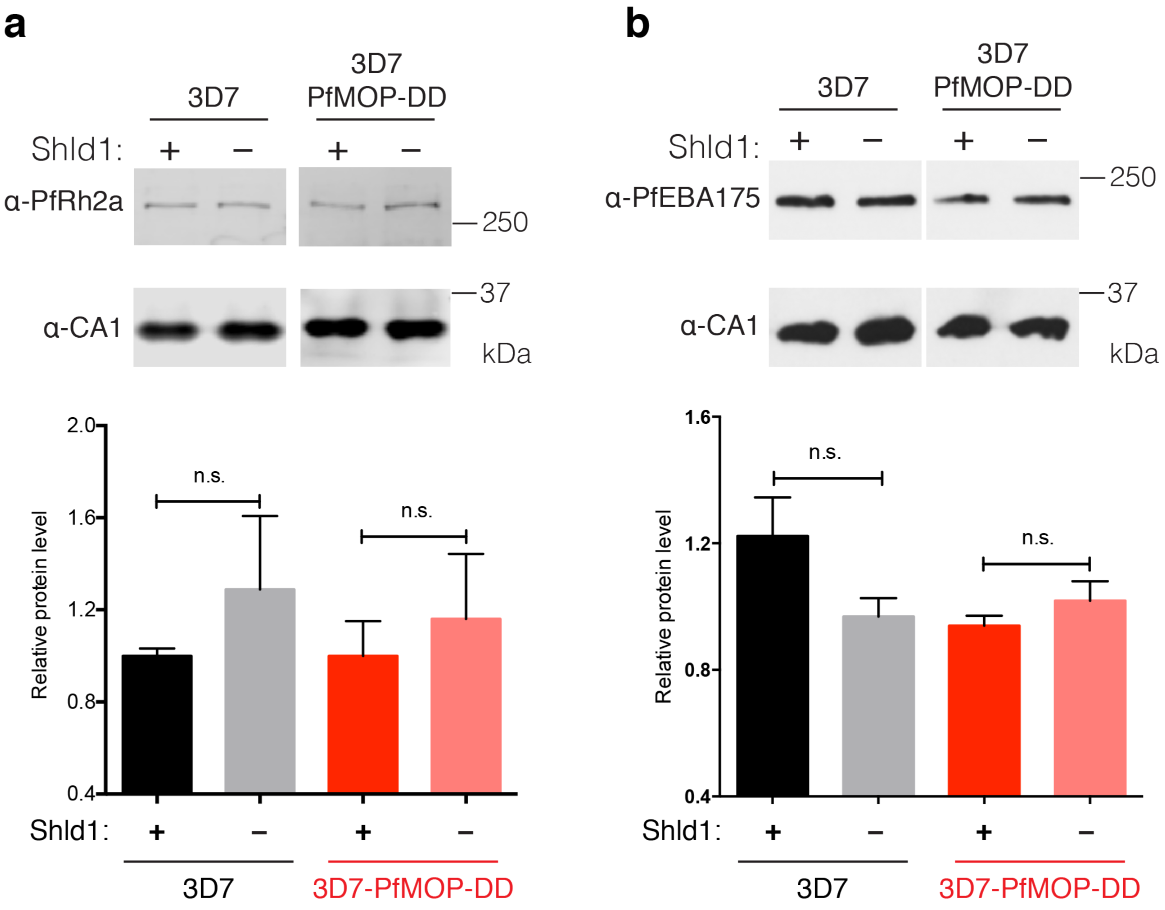

Supplementary Fig. 12. Microneme and rhoptry protein release into culture supernatants. 3D7-PfMOP-DD or the parental 3D7 parasites were treated with chymotrypsin (1 mg/ml), trypsin (1 mg/ml), and neuraminidase (66.7 mU/ml) to prevent reinvasion, grown with or without Shld1 until schizont rupture was complete, and supernatants were harvested and clarified by centrifugation. Immunoblots were performed with (**a**) anti-PfEBA175 and (**b**) anti-Rh2a to quantify release of these proteins from micronemes and rhoptries, respectively (upper panels). Antibodies to red blood cell carbonic anhydrase-1 (CA-1) were used to normalize for RBC rupture. The relative amount of each protein was quantified with the LiCor Odyssey CLx system (lower panels) (n=3-7 biological replicates, mean with SEM).

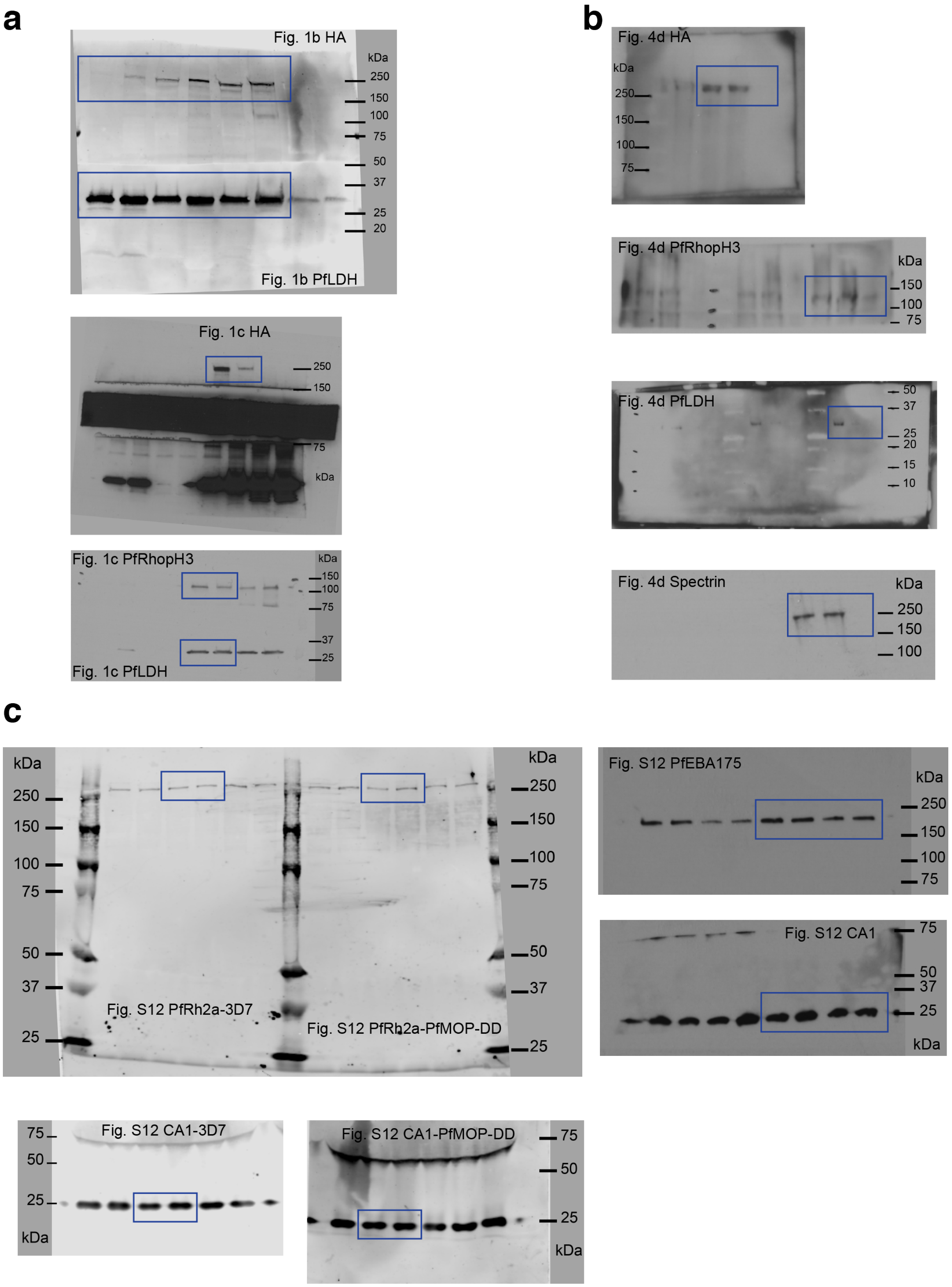

Supplementary Fig. 13. Full-sized images of all immunoblots. Full-sized immunoblots from

figure 1 (**a**), figure 4 (**b**), and supplementary figure 12 (**c**) are shown. The blue box on each immunoblot shows the cropped area displayed in the other figures.
